# Molecular Signature of Astrocytes for Gene Delivery by the Synthetic Adeno‐Associated Viral Vector rAAV9P1

**DOI:** 10.1002/advs.202104979

**Published:** 2022-04-10

**Authors:** Amelie Bauer, Matteo Puglisi, Dennis Nagl, Joel A Schick, Thomas Werner, Andreas Klingl, Jihad El Andari, Veit Hornung, Horst Kessler, Magdalena Götz, Dirk Grimm, Ruth Brack‐Werner

**Affiliations:** ^1^ Institute of Virology Helmholtz Center Munich Neuherberg 85764 Germany; ^2^ Physiological Genomics Biomedical Center (BMC) Ludwig‐Maximilians‐Universität (LMU) Planegg‐Martinsried 82152 Germany; ^3^ Institute for Stem Cell Research Helmholtz Center Munich Biomedical Center (BMC) Ludwig‐Maximilians‐Universität (LMU) Planegg‐Martinsried 82152 Germany; ^4^ Gene Center and Department of Biochemistry Ludwig‐Maximilians‐Universität Munich 81377 Germany; ^5^ Institute of Molecular Toxicology and Pharmacology Genetics and Cellular Engineering Group Helmholtz Center Munich Neuherberg 85764 Germany; ^6^ Department of Computational Medicine and Bioinformatics & Department of Internal Medicine University of Michigan Ann Arbor MI 48109 USA; ^7^ Plant Development and Electron Microscopy Department Biology I Biocenter Ludwig‐Maximilians‐Universität (LMU) Planegg‐Martinsried 82152 Germany; ^8^ BioQuant Center and Cluster of Excellence CellNetworks at Heidelberg University Heidelberg 69120 Germany; ^9^ Department of Infectious Diseases Virology Medical Faculty Heidelberg University Heidelberg 69120 Germany; ^10^ Institute for Advanced Study and Center of Integrated Protein Science (CIPSM) Department Chemie Technische Universität München Garching 85748 Germany; ^11^ Excellence Cluster of Systems Neurology (SYNERGY) Munich 81377 Germany; ^12^ German Center for Infection Research (DZIF) and German Center for Cardiovascular Research (DZHK) Partner site Heidelberg Heidelberg 69120 Germany; ^13^ Department of Biology II Ludwig‐Maximilians‐Universität (LMU) Planegg‐Martinsried 82152 Germany

**Keywords:** AAV, Adeno‐associated virus, astrocytes, integrins, receptor profile, vectors

## Abstract

Astrocytes have crucial functions in the central nervous system (CNS) and are major players in many CNS diseases. Research on astrocyte‐centered diseases requires efficient and well‐characterized gene transfer vectors. Vectors derived from the Adeno‐associated virus serotype 9 (AAV9) target astrocytes in the brains of rodents and nonhuman primates. A recombinant (r) synthetic peptide‐displaying AAV9 variant, rAAV9P1, that efficiently and selectively transduces cultured human astrocytes, has been described previously. Here, it is shown that rAAV9P1 retains astrocyte‐targeting properties upon intravenous injection in mice. Detailed analysis of putative receptors on human astrocytes shows that rAAV9P1 utilizes integrin subunits *α*v, *β*8, and either *β*3 or *β*5 as well as the AAV receptor AAVR. This receptor pattern is distinct from that of vectors derived from wildtype AAV2 or AAV9. Furthermore, a CRISPR/Cas9 genome‐wide knockout screening revealed the involvement of several astrocyte‐associated intracellular signaling pathways in the transduction of human astrocytes by rAAV9P1. This study delineates the unique receptor and intracellular pathway signatures utilized by rAAV9P1 for targeting human astrocytes. These results enhance the understanding of the transduction biology of synthetic rAAV vectors for astrocytes and can promote the development of advanced astrocyte‐selective gene delivery vehicles for research and clinical applications.

## Graphical abstract

Recombinant Adeno‐associated virus vector 9P1 (rAAV9P1) is a peptide‐displaying variant of AAV9 that targets human (in vitro) and murine (in vivo) astrocytes with high efficiency. rAAV9P1 utilizes the integrins *α*v*β*8 and αvβ3 or αvβ5, glycan residues and the AAV receptor (AAVR) to transduce astrocytes. Various astrocyte‐relevant intracellular pathways also contribute to efficient transduction of astroyctes by rAAV9P1.

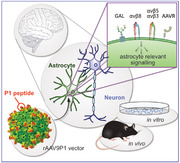

## 1 Introduction

Astrocytes are a major group of glial cells in the central nervous system (CNS) that represent an estimated total number of over 15 billion cells in the human brain.^[^ ^1^, ^2^ ^]^ Astrocytes have diverse roles in many crucial functions of the CNS.^[^ ^3^, ^4^ ^]^ These include support and regulation of the blood–brain barrier (BBB), communication with neurons and other astrocytes via Ca^2+^ signaling, as well as brain development. Astrocytes are essential for the functioning of neurons and the formation, functioning, plasticity, and elimination of synapses.^[^ ^5^, ^6^ ^]^ They maintain the homeostasis of crucial ions (e.g., K^+^) and the pH of the synaptic interstitial fluid and clear excitatory and inhibitory neurotransmitters from synaptic spaces. Furthermore, astrocytes provide neurons with energy metabolites and substrates for the production of neurotransmitters.^[^ ^7^ ^]^

In addition, astrocytes are essential for nervous system responses to injuries, including formation of glial scars and neuroinflammation.^[^ ^8^ ^]^ Pathological changes of astrocytes occur in several diseases of the CNS, including Alzheimer's disease, Huntington's disease, Parkinson's disease, amyotrophic lateral sclerosis, and Alexander disease.^[^ ^8^, ^9^ ^]^ Finally, astrocytes and astrocyte‐like neural stem cells can give rise to primary brain tumors, i.e., glioblastomas.^[^ ^10^, ^11^ ^]^ Glioblastomas are among the most aggressive neoplasms and typically come with a poor prognosis, with a median survival of around 15 months.

Because of the debilitating nature and fatal outcome of these CNS diseases, the development of therapeutic modalities has high priority. In particular, there is an urgent need for gene delivery vehicles for targeting astrocytes and for a profound understanding of the molecular nature of their interactions with astrocytes. The nonpathogenic adeno‐associated viruses (AAV) are nonenveloped viruses with single‐stranded DNA genomes of ≈4.7 kb. Thirteen serologically distinct AAV serotypes and numerous variants have been isolated from human and nonhuman tissues. AAV‐based recombinant vectors (rAAV) have numerous advantages for gene delivery,^[^ ^12^, ^13^, ^14^, ^15^ ^]^ including their ability to transduce dividing and nondividing cells as well as the long‐term persistence of episomal rAAV genomes in nondividing cells with very low integration rates.^[^ ^16^ ^]^ In addition to their widespread use as research tools, evaluation of data from 149 clinical trials (until 2019) with more than 3000 patients overall supports safety and tolerability of rAAV vectors in humans.^[^ ^17^ ^]^ The most frequently used rAAV vectors in these studies contained the capsid from the AAV2 serotype because of its wide tissue and cellular tropisms. However, over recent years, there has been an increase in clinical studies using vectors with capsids derived from AAV9, mainly for gene delivery to the CNS. rAAV9 vectors have been shown to cross the BBB and transduce glial cells and neurons in the brain of rodents and nonhuman primates.^[^ ^18^, ^19^, ^20^, ^21^ ^]^ An example for its applicability in humans is the recent approval of the rAAV‐based gene therapy Zolgensma that delivers a functional copy of the *SMN1* gene to spinal motor neurons of patients with spinal muscular atrophy.^[^ ^22^ ^]^ Therefore, rAAV9 is a prototype of brain‐targeting rAAV vectors.

Interactions of AAV capsid proteins with molecules on host cell surfaces are crucial determinants of cell and tissue specificity of rAAV vectors. Studies with different AAV serotypes have identified many cell surface molecules that influence the attachment and entry of AAV capsids.^[^ ^13^, ^23^ ^]^ These include a “universal” AAV receptor (AAVR) utilized by at least seven serotypes including AAV2 and AAV9. Additional host factors that influence transduction properties of rAAV vectors include glycans, such as heparan sulfate proteoglycans (HSPG), sialic acid, and terminal N‐linked galactose.^[^ ^24^, ^25^ ^]^

In a previous study, we showed that an AAV9‐derived vector (rAAV9P1), that displays a foreign, seven amino acids‐long peptide (P1, sequence RGDLGLS), efficiently transfers genes to human astrocytes.^[^ ^26^ ^]^ Here, we reveal the molecular signature of astrocytes enabling transduction by rAAV9P1 and validate astrocyte‐targeting and selectivity of rAVV9P1 in the brain in vivo. These results shed light on the biology of a synthetic rAAV capsid and establish rAAV9P1 as a prototypical astrocyte‐targeting AAV vector.

## 2 Results

### 2.1 rAAV9P1 Vectors Transduce Human Astrocytes more Efficiently than rAAV9 and with Higher Selectivity than rAAV2

In a previous publication, we showed that rAAV9P1 efficiently transduces astrocytic cell lines (i.e., HNSC.100 and U251MG) as well as primary and induced pluripotent stem cell (iPSC)‐derived astrocytes and primary human astrocytes with high efficiencies, while primary and iPSC‐derived neurons were only marginally transduced.^[^ ^26^ ^]^ In agreement, rAAV9P1 vectors have been reported to show very weak transduction of the neuroblastoma cell line SH‐SY5Y.^[^ ^27^ ^]^ In contrast, SH‐SY5Y cells were efficiently transduced by rAAV2 vectors^[^ ^27^ ^]^ which are known to have broad cell tropism.^[^ ^28^ ^]^ To further investigate their discrepant transduction behaviors, we directly compared the astrocyte‐transducing potential of rAAV9P1 capsids with that of rAAV2 and of rAAV9, from which rAAV9P1 was derived.^[^ ^26^ ^]^ We used vectors containing self‐complementary (sc) AAV genomes with an enhanced yellow fluorescent protein (eYFP) reporter driven by the cytomegalovirus (CMV) promoter.^[^ ^29^ ^]^ In contrast to wildtype (WT) AAVs, scAAVs carry an inverted repeat genome that forms a double‐stranded genome upon entry into the cell and therefore allows for quick onset of transcription without the need for DNA synthesis or annealing of separate plus or minus DNA strands.^[^ ^30^ ^]^ In addition to HNSC.100 and U251MG cells as astrocytic cell lines, we included commonly used nonbrain human cell lines (i.e., HeLa and ek293T cell lines) as models for nonastrocytic cells. Transduction efficiencies of the rAAV vectors were evaluated by flow cytometry.

**Figure 1:**
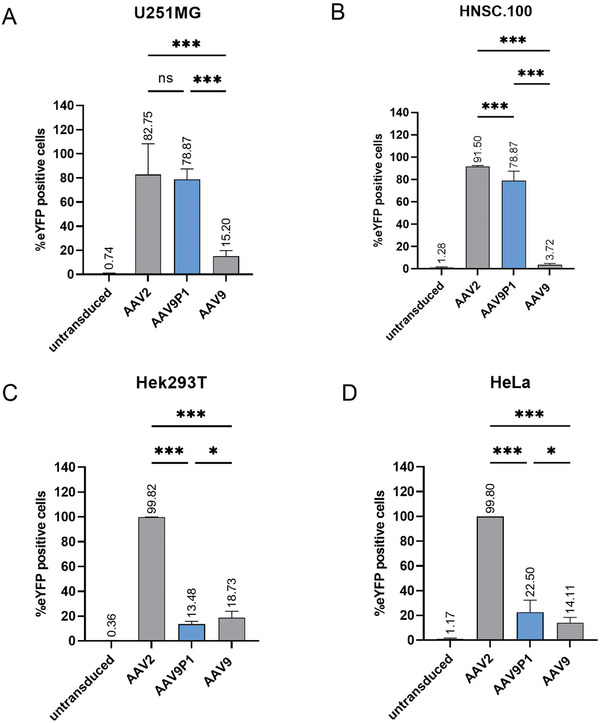
Efficiency and selectivity of rAAV9P1 vectors in cultured human cells, compared to rAAV2 and rAAV9 vectors. Cultures of human astrocytic cell lines (U251MG (A) and HNSC.100 (B)) or nonastrocytic cell lines (Hek293T (C) and HeLa (D)) were exposed to rAAV9P1, rAAV2, or rAAV9 vectors. Vector particles contained scAAV genomes with a transgene for expression of enhanced yellow fluorescent protein (eYFP). Cultures were analyzed by flow cytometry 48 h after exposure to vector and percentages of eYFP‐positive cells were determined. Absolute values are depicted above the respective bar. Data are presented as mean ± SEM (*n* = 3). *p*‐values are calculated using one‐way ANOVA with Šidák correction. ns (nonsignificant) *p* > 0.05, *p ≤ 0.05, ***p ≤ 0.001.

As shown in **Figure** **1**, rAAV9P1 vectors transduced the astrocytic cell lines with high efficiencies (>75%, Figure 1A,B), whereas they showed much lower transduction efficiencies for the nonastrocytic cell lines (<25%, Figure 1C,D). In contrast, rAAV2 vectors displayed similarly high transduction efficiencies for all tested cell lines, indicating that rAAV2 vectors fail to discriminate between the astrocytic and non‐glial human cells. rAAV9 vectors showed low transduction efficiencies in human astrocytic as well as non‐glial cells without selectivity for the astrocytic cells (<20%, Figure 1A–D). Together, these results show that rAAV9P1 vectors display reduced cell tropism compared to rAAV2 vectors and increased astrocyte‐targeting properties compared to the parental rAAV9 vectors.

### 2.2 Targeting of Astrocytes in Mouse Brain by rAAV9P1 after Tail Vein Injection

It is well‐known that transduction efficiencies and specificities of rAAV vectors can vary between species^[^ ^31^, ^32^, ^33^, ^34^ ^]^ as well as between in vitro and in vivo settings.^[^ ^35^, ^36^, ^37^, ^38^, ^39^ ^]^ To test whether rAAV9P1 can be used for selective targeting of astrocytes in vivo in mice, we investigated the CNS transduction pattern of rAAV9P1 after injection into the lateral tail vein of adult C57BL6/J mice. One month after injection of 2–3 × 10^12^ rAAV9P1 vector genomes (vg) per mouse, mice were perfused with phosphate‐buffered saline (PBS) and paraformaldehyde, and brains were collected for sectioning. Transduction was analyzed by immunostaining of the reporter protein encoded by the transduced gene (eYFP or green fluorescent protein (GFP)) and quantification of around 900 cells in total.

**Figure 2:**
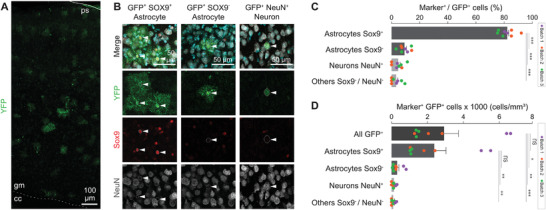
Targeting of brain astrocytes by rAAV9P1 vectors in mice after tail vein injection. A) Confocal micrographs showing an overview of the mouse motor cortex 1 month after the systemic injection (months post‐injection = mpi) of rAAV9P1. eYFP expression was detected using an anti‐GFP antibody that cross‐reacts with eYFP (cc: corpus callosum; gm: gray matter; ps: pial surface). B) Confocal micrographs showing the transduction of Sox9^+^ or Sox9^−^ astrocytes, as well as NeuN^+^ neurons. C+D) Histograms showing the transduction rates of different cell types. Data are shown for injections of three rAAV9P1 vector batches. Batch 1 (purple): rAAV9P1‐CMV‐eYFP, 1 mpi, intact cortex; Batch 2 (orange): rAAV9P1‐CMV‐GFP, 1 mpi, intact cortex, contralateral to stab wound injury; Batch 3 (green): rAAV9P1‐CMV‐eYFP, 1 mpi, intact cortex. Dots indicate values for individual animals, colors of dots signify the vector batch used for injections. Data are presented as mean ± SEM (*n* = 8). *p*‐values are calculated using C) one‐way ANOVA or D) Kruskal–Wallis test. ns *p* > 0.05, *p ≤ 0.05, **p ≤ 0.01, ***p ≤ 0.001. Scale bars represent A) 100 µm and B) 50 µm.

In sections of the murine cerebral cortex, cells positive for reporter gene expression consisted mainly of bushy cells with astrocyte morphology and were apparent in all the cortical layers (**Figure** **2**A). Quantification of transduced cells in the gray matter throughout all layers showed that most of the transduced cells are positive for the astrocyte‐specific nuclear marker Sox9^+^ (Astrocytes Sox9^+^, 82.3% ± 5.6; Figure 2B,C).^[^ ^40^ ^]^ This indicated that the main target cells of rAAV9P1 in the mouse cerebral cortex gray matter are protoplasmic, Sox9^+^ astrocytes (Figure 2B,C). Notably, rAAV9P1 also targeted a smaller population of cells with clear astrocyte morphology that did not have detectable levels of Sox9 immunostaining signal (Astrocytes Sox9^−^; 9.3% ± 3.9, Figure 2B,C). Conversely, a very low percentage of reporter‐positive cells was neurons as identified by morphology and staining for the neuron‐specific marker NeuN^[^ ^2^ ^]^ (indicated by NeuN^+^), or other cells that did not stain for either marker (indicated by Sox9^−^/NeuN^−^; 8.0% ± 6.6). Quantification of absolute cell numbers revealed 2922 ± 2275 fluorophore‐positive cells per mm^3^, of which 2691 ± 2115 cells were astrocytes (Figure 2D). Thus, i.v. injection of rAAV9P1 resulted in highly specific expression of the vector‐delivered CMV promoter‐driven reporter gene in astrocytes of the mouse cerebral cortex gray matter in intact brains.

Initial results from a study investigating transduction behavior of rAAV9P1 in a murine stab wound model showed that systemically (i.e., tail vein) injected rAAV9P1 vectors were able to invade stab wound lesions and to transduce astrocytes in these (Figure S1, Supporting Information). This indicates that the astrocyte‐transducing capacity of rAAV9P1 in mice can be extended to injured brain tissues and is not limited to intact brain tissues.

### 2.3 rAAV9P1 Requires Selected RGD‐Binding Integrins for Transduction of Human Astrocytes

**Figure 3:**
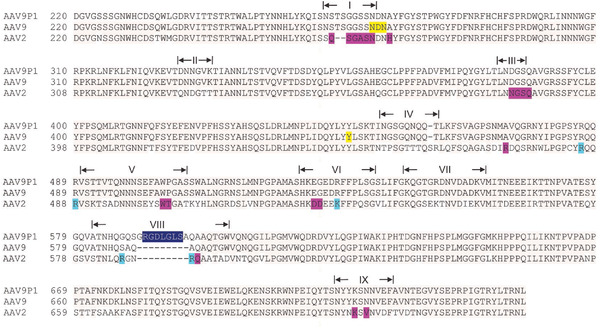
Position of the P1 peptide in the VP3 protein sequence of the rAAV9P1 capsid. Alignment of the sequences of Viral Protein 3 (VP3) of rAAV9P1, AAV9, and AAV2 capsids. Reference for amino acid numbering is the first amino acid at the N‐terminus of Viral Protein 1 (VP1). Roman numerals indicate variable regions (VR) of AAV9 according to DiMattia et al.^[^ ^41^ ^]^ The P1 peptide (highlighted in dark blue) is located in VR‐VIII. Galactose binding sites of AAV9 are highlighted in yellow,^[^ ^43^ ^]^ the predicted AAVR‐binding residues of AAV2 in magenta,^[^ ^44^ ^]^ and heparan sulfate proteoglycan (HSPG)‐binding amino acids in light blue.^[^ ^45^ ^]^ Gray background color indicates sequence identity. Mismatches between sequences are indicated by white background.

Given the capacity of rAAV9P1 for transduction of both, human and mouse astrocytic cells, we investigated the underlying molecular signature of astrocytes responsible for transduction with rAAV9P1, using human astrocytes. To identify receptors that promote the interaction of the rAAV9P1 capsid with astrocytes and their subsequent transduction, we focused on possible binding partners of the P1 peptide. As depicted in **Figure** **3**, the P1 peptide sequence is the only difference between the major capsid protein VP3 of rAAV9P1 and its parental serotype AAV9. The insertion site of the peptide between Q_585_ and A_586_ of VP3_AAV9_ was chosen because of its exposed position within the assembled capsid on the tip of the protrusions (variable region VIII) on the capsid surface (Figure S2B, Supporting Information).^[^ ^38^, ^41^ ^]^ Bioinformatics predictions also suggest an increase in size and flexibility of the loop harboring the P1 sequence in VP3_AAV9P1_, compared to this region in VP3_AAV9_ (Figure S2A, Supporting Information). Electron microscopy analysis of rAAV9P1 vector particles purified by iodixanol density gradient revealed no significant differences in particle size or gross morphological alterations compared to the natural serotypes AAV2 and AAV9 (Figure S2C,D, Supporting Information). Capture of finer structural features of AAV capsids by cryo‐electron microscopy and image reconstruction was previously reported to be very difficult.^[^ ^42^ ^]^

The tri‐peptide sequence RGD, which is part of the P1 peptide of rAAV9P1, is an integrin recognition motif contained in many integrin ligands, including viral surface proteins and proteins of the extracellular matrix.^[^ ^46^, ^47^, ^48^, ^49^ ^]^ Integrins bind to their ligands as heterodimeric transmembrane proteins that consist of an *α*‐ and a *β*‐subunit. RGD‐binding integrin heterodimers include *α*v*β*1, *α*v*β*3, *α*v*β*5, *α*v*β*6, *α*v*β*8, *α*5*β*1, *α*IIb*β*3, and *α*8*β*1.

**Figure 4:**
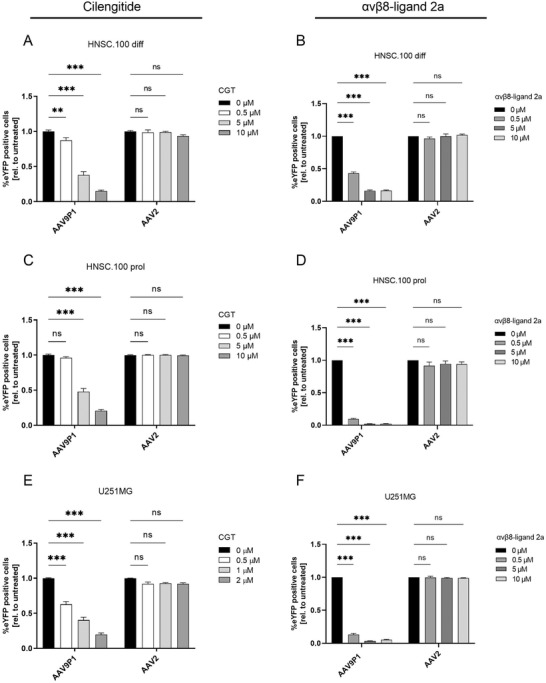
Influence of ligands of the RGD‐binding integrins *α*v*β*5 and *α*v*β*3 (Cilengitide) or *α*v*β*8 (*α*v*β*8‐ligand 2a) on transduction of human astrocytes by rAAV9P1. A+C+E) Inhibition of rAAV9P1 transduction by Cilengitide (CGT) treatment. B+D+F) Inhibition of rAAV9P1 transduction by *α*v*β*8‐ligand 2a treatment. Cultures of differentiated HNSC.100 astrocytes (A+B) HNSC.100 diff), proliferating HNSC.100 astrocyte progenitors (C+D, HNSC.100 prol), and E+F) U251MG astrocytic glioblastoma cells were exposed simultaneously to integrin peptide ligands (CGT or *α*v*β*8‐ligand 2a) and rAAV vectors (rAAV9P1‐eYFP or rAAV2‐eYFP) for 48 h before transgene expression was analyzed by flow cytometry. Data are presented as mean ± SEM and are presented relative to vector‐exposed, untreated control cells (*n* = 3). *p*‐values are calculated using two‐way ANOVA with Šidák correction. ns *p* > 0.05, **p ≤ 0.01, ***p ≤ 0.001.

Evaluation of publicly available expression data of human astrocytes and neurons^[^ ^50^ ^]^ showed expression of integrins *α*v*β*3, *α*v*β*5, and *α*v*β*8 in human and in murine astrocytes, both from healthy and injured brains and in human astrocytic cell lines, including U251MG (Figure S3A–C, Supporting Information). Therefore, we investigated the effects of small‐molecule ligands of RGD‐binding integrins on the transduction of human astrocytes by rAAV9P1. rAAV2 vectors were assessed for comparison. Cilengitide (CGT; cRGDfNMeV) is a cyclic penta‐peptide that selectively binds to *α*v*β*3 and *α*v*β*5 integrins.^[^ ^51^ ^]^ Treatment with nontoxic CGT concentrations dose‐dependently inhibited rAAV9P1 transduction of cells in HNSC.100 differentiated and proliferating astrocyte cultures and in U251MG cultures (**Figure** **4**A,C,E; cell viability data in Figure S4A,C,E, Supporting Information). Transduction efficiencies of rAAV9P1 decreased to ≤ 20% of untreated cells at the highest CGT concentrations. In contrast to rAAV9P1, CGT treatment did not affect transduction with rAAV2 vectors. To block *α*v*β*8, we used a cyclic octa‐peptide (*α*v*β*8‐ligand 2a; c(GLRGDLp(NMe)K(Ac))) that binds *α*v*β*8 with high affinity and high selectivity.^[^ ^52^ ^]^ Treatment with nontoxic concentrations of *α*v*β*8‐ligand 2a strongly and dose‐dependently inhibited the transduction of HNSC.100 astrocytic cells (differentiated and proliferating cells) and U251MG cells by rAAV9P1 vectors (Figure 4B,D,F; cell viability data in Figure S4B,D,F, Supporting Information). At the highest concentrations of *α*v*β*8‐ligand 2a, transduction efficiencies decreased to ≤ 5% of untreated cells. Transduction by rAAV2 vectors was not affected. This shows that CGT and *α*v*β*8‐ligand 2a treatment selectively impairs the transduction of astrocytes with rAAV9P1 but not with rAAV2.

**Figure 5:**
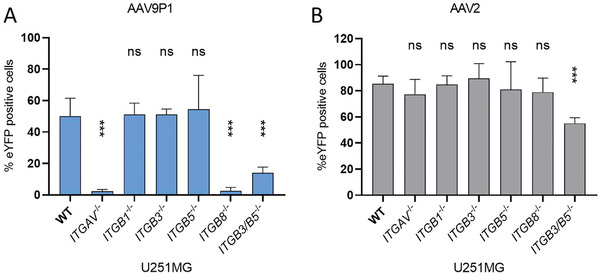
Inhibition of transduction of U251MG cells by rAAV9P1 vectors by CRISPR/Cas9‐mediated knockout of *αv*, *β5+β3*, or *β8* integrin subunits. U251MG WT and U251MG integrin subunit knockout cells (U251MG *ITGAV* ^−/−^, *ITGB1* ^−/−^, *ITGB3* ^−/−^, *ITGB5* ^−/−^, *ITGB8* ^−/−^, *ITGB3/B5* ^−/−^) were transduced with A) rAAV9P1‐eYFP or B) rAAV2‐eYFP vectors. Transgene expression was analyzed 48 h post‐transduction by flow cytometry. Data are presented as mean ± SEM and are presented relative to WT control cells (*n* = 3). *p*‐values are calculated using one‐way ANOVA with Šidák correction. ns *p* > 0.05, ***p ≤ 0.001.

To further validate the importance of RGD‐binding integrins for astrocyte transduction by rAAV9P1, we generated U251MG knockout cell lines for selected RGD‐binding integrin subunits by CRISPR/Cas9‐mediated genome editing. Single‐guide RNAs (sgRNAs) were designed to target *ITGAV*, *ITGB1*, *ITGB3*, *ITGB5*, and *ITGB8* genes (sequences in Table S1, Supporting Information) to eliminate expression of the integrin subunits *α*v, *β*1, *β*3, *β*5, and *β*8, respectively. Knockouts were validated by sequence analysis. In addition, levels of surface expression of the assembled heterodimers or individual integrin subunits were analyzed by surface staining (Figure S5A, Supporting Information). Knockout of the integrin *α*v subunit (*ITGAV^−/−^*), preventing the formation of all *α*v‐containing integrin heterodimers, resulted in a complete loss of transduction by rAAV9P1 (**Figure** **5**A). In addition, knockout of the *β*8 subunit alone (*ITGB8^−/−^*) also decreased rAAV9P1 transduction by almost 100%. Interestingly, single knockouts of the subunits *β*1 (*ITGB1^−/−^*), *β*3 (*ITGB3^−/−^*), or *β*5 (*ITGB5^−/−^*) did not affect transduction by rAAV9P1. To mimic the treatment of cells with CGT, we generated U251MG cell lines with knockouts of both, the *β*3 and *β*5 subunits (*ITGB3/B5* ^−/−^). Transduction efficiencies of these double knockout cells by rAAV9P1 were reduced to less than 30% of parental cells. In contrast, transduction with rAAV2 was affected only in the *β*3 and *β*5 double knockout cells (*ITGB3/B5* ^−/−^), and reduction was moderate, although significant (Figure 5B).

Together, these results demonstrate that the subunits *α*v and *β*8, as well as the combination of subunits *β*3 and *β*5, are crucial for transduction of human astrocytes by rAAV9P1.

### 2.4 rAAV9P1 Requires AAVR and N‐Linked Terminal Galactose for Efficient Transduction of Human Astrocytes

**Figure 6:**
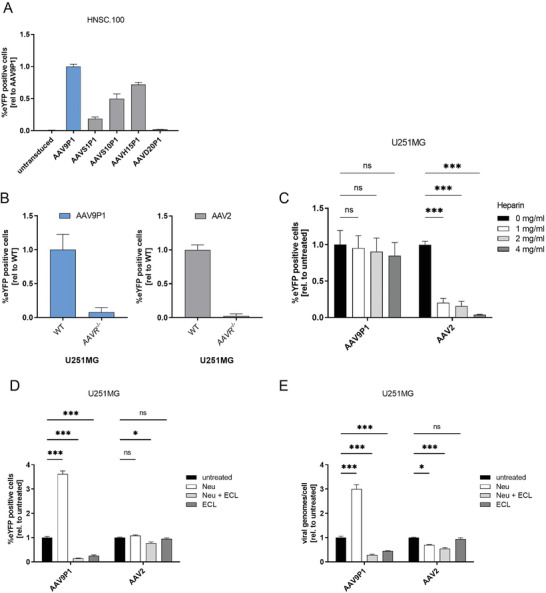
Evaluation of the importance of nonintegrin AAV receptors for transduction of human astrocytes by rAAV9P1. A) HNSC.100 astrocytic progenitor cells (HNSC.100 prol) were transduced with rAAV9P1‐eYFP and four rAAV P1 variants (rAAVS1P1, rAAVS10P1, rAAVH15P1, rAAVD20P1) carrying the P1 peptide at the same location in the VP3 protein as rAAV9P1. Transgene expression was measured 48 h post‐transduction by flow cytometry. B) WT U251MG or U251MG knockout cells for the AAVR receptor (*AAVR^−/−)^* were transduced with rAAV9P1‐eYFP or rAAV2‐eYFP. Transgene expression was analyzed 48 h post‐vector exposure by flow cytometry. C) Heparin‐competition assays were performed by co‐incubating U251MG cells with various concentrations of soluble heparin and rAAV9P1‐eYFP or rAAV2‐eYFP vectors for 4 h at 37 °C. Transgene expression was analyzed 48 h post‐transduction by flow cytometry. D+E) U251MG cells were treated with neuraminidase for the removal of sialic acids (Neu). N‐linked glycans on U251MG cells were blocked by incubation with *Erythrina cristagalli* lectin (ECL). ECL incubations were performed on cells pretreated with neuraminidase (Neu + ECL) or with untreated cells (ECL). Cultures were exposed to rAAV9P1‐eYFP or rAAV2‐eYFP vectors. Cultures were analyzed for D) transgene expression by FACS analysis and E) for binding of rAAV particles to cells by qRT‐PCR via recombinant AAV binding assay (rABA). Data are presented as mean ± SEM and are presented relative to cells transduced with A) rAAV9P1, B) WT cells, or C–E) vector‐exposed, untreated control cells (*n* = 3). *p*‐values are calculated using two‐way ANOVA with Šidák correction. ns *p* > 0.05, *p ≤ 0.05, ***p ≤ 0.001.

To determine whether the P1 peptide is sufficient to govern the efficient transduction of human astrocytes by rAAV vectors, we assayed the transduction behavior of four additional rAAV variants containing the P1 peptide (rAAVS1P1, rAAVS10P1, rAAVH15P1, and rAAVD20P1). All variants have been previously selected for efficient transduction of various non‐glial cell types in mice (skeletal muscle, heart, diaphragm; data from El Andari et al., manuscript in revision), confirming their transduction competence. All variants transduced HNSC.100 cells with lower efficiencies than rAAV9P1 (**Figure** **6**A), indicating that the P1 peptide alone is not sufficient for efficient transduction of human astrocytes. To identify additional receptors required for transduction of astrocytes by rAAV9P1, we investigated the importance of several known AAV host cell receptors or co‐receptors for the transduction of human astrocytes by rAAV9P1. The most broadly used receptor by AAV serotypes is the KIAA0319L protein, also known as AAVR.^[^ ^23^, ^25^ ^]^ Knockout of AAVR in U251MG cells completely abrogated transduction with rAAV9P1 as well as rAAV2 (Figure 6B), confirming that rAAV9P1, like rAAV2 vectors, utilize AAVR to transduce U251MG cells (also see Figure S5B, Supporting Information).

Glycan receptors used by AAVs include HSPG, sialic acids, and N‐linked surface glycans with terminal galactose.^[^ ^53^ ^]^ To investigate the role of HSPG, we performed transduction experiments in the presence of different concentrations of soluble heparin. These heparin‐competition experiments showed that transduction of U251MG cells with rAAV9P1 was not affected by heparin, while transduction with rAAV2 was strongly inhibited under the same conditions (Figure 6C). These results indicate that transduction of U251MG cells by rAAV9P1 does not depend on HSPG.

To investigate the role of sialic acids, cells were treated with neuraminidase (NEU, from *Vibrio cholerae*) to remove terminal sialic acids from cell‐surface glycans. NEU treatment increased the transduction efficiency and virus binding of rAAV9P1 to astrocytes (Figure 6D,E). No effect was observed on rAAV2 transduction of astrocytes. This indicates that sialic acids on the cell surface of astrocytes do not act as receptors for rAAV9P1 but rather hamper rAAV9P1 transduction. This was reported previously for rAAV9, which was shown to require terminal galactose for transduction.^[^ ^54^ ^]^ To investigate the importance of terminal galactosyl‐residues for rAAV9P1 transduction of astrocytes, cells were treated with ECL, a lectin from *Erythrina cristagalli*, that specifically binds to galactose residues, in particular Gal(*β*1,4)N‐GlcNAc.^[^ ^55^ ^]^ Treatment with ECL strongly reduced transduction of U251MG cells by rAAV9P1 (Figure 6D) and binding of vector particles to the cells (Figure 6E). Transduction and vector binding by rAAV2 were not significantly affected. These results indicate that rAAV9P1 uses N‐linked terminal galactose for transduction of astrocytes. As a whole, these results demonstrate that rAAV9P1 transduction is dependent on the presence of the co‐receptors AAVR and N‐linked terminal galactose.

### 2.5 Transduction of U251MG Cells by rAAV9P1 Involves Astrocyte‐Relevant Intracellular Pathways

**Figure 7:**
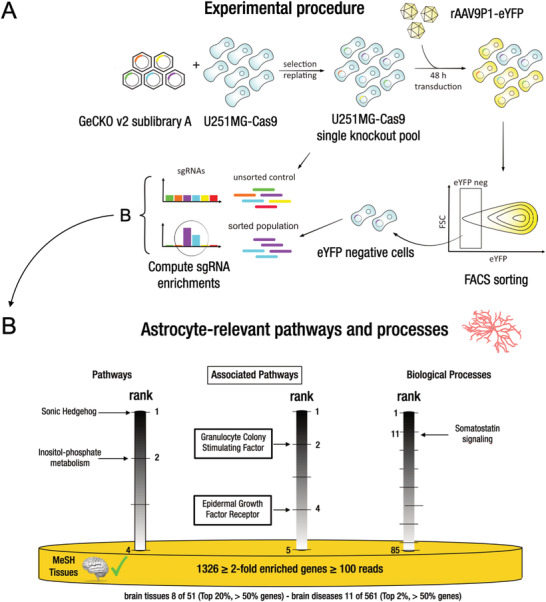
Intracellular astrocyte‐relevant pathways required for transduction by rAAV9P1 identified by unbiased genome‐wide CRISPR/Cas9 knockout screening of U251MG cells. A) Experimental workflow for genome‐wide CRISPR/Cas9 genome knockout screening of U251MG cells. U251MG cells stably expressing Cas9 (U251MG‐Cas9) were transduced with a lentiviral human GeCKOv2 sgRNA library A^[^ ^56^ ^]^ and subjected to antibiotic selection to establish the single‐knockout cell pool (U251MG‐Cas9_GeCKO). The U251MG‐Cas9_GeCKO cell pool was exposed to rAAV9P1‐eYFP vectors and eYFP‐negative cells selected by FACS sorting. Genomic DNA was isolated from this eYFP‐negative subpopulation and the U251MG‐Cas9_GeCKO cell pool (= unsorted control) and sgRNA sequences were identified by NGS. sgRNA sequences significantly enriched in the eYFP‐negative subpopulation compared to the unsorted control were used to identify an initial set of 2715 genes with enriched corresponding sgRNAs in the eYFP‐negative cell subpopulation. From this initial set, a final set of 1326 genes was extracted by more stringent selection criteria (identification by ≥ 100 reads and ≥ 2‐fold enrichment) and used for GO‐term analysis. B) GO‐term and pathway analysis reveals enrichment of astrocyte‐relevant pathways and biological processes in the eYFP‐negative subpopulation. The yellow disk represents the final set of 1326 genes (≥ 2‐fold‐enriched sgRNAs, ≥ 100 reads) and the strong brain and brain disease association of this gene set (MeSH terms and tissues). The vertical grayscales indicate rank position of the astrocyte‐relevant terms within the total number of associated terms for pathways/associated pathways (left) and biological processes (right, only top 30% included).

Having elucidated the surface receptor requirements of rAAV9P1, we next investigated whether intracellular pathways and processes with known relevance in astrocytes are also involved in transduction by rAAV9P1. To this end, we used an unbiased approach entailing CRISPR/Cas9 genome‐wide knockout screening in U251MG cells as outlined in **Figure** **7**A. We then purified cells that were not permissive for transduction by rAAV9P1 and identified preferentially knocked out genes presumptively knocked out in this subpopulation compared to controls. Briefly, U251MG cells stably expressing Cas9 were transduced with a pooled lentiviral human CRISPR sgRNA library (GeCKO v2 sublibrary A^[^ ^56^ ^]^) and subsequently exposed to rAAV9P1‐eYFP.

The eYFP‐negative subpopulation was isolated by flow cytometry sorting, and sgRNA sequences over‐represented in this population in comparison to an unsorted control population were amplified from genomic (g)DNA, sequenced, and deconvolved using ENCoRE software.^[^ ^57^ ^]^ A final set of 1326 genes showing an average fold‐change of 10.23 (range: 2.0–148) of the enriched corresponding sgRNAs was filtered from an initial set of 2715 gene candidates (for details, see Experimental Section and Figure S6 and Table S2, Supporting Information) and subjected to pathway, GO‐, and MeSH‐Term, as well as tissue enrichment analysis using the GeneRanker tool.

The results are summarized in Figure 7B. MeSH‐terms and tissue association confirmed that brain tissues and diseases ranked very close to the top of the respective lists of associated terms and included over 50% of the identified genes.

In total, the analysis identified five canonical and four associated pathways preferentially knocked out in the eYFP‐negative population. Four of these pathways (two canonical, two associated) and one biological process were found to be relevant for astrocytes (Figure 7B). These are pathways involving sonic hedgehog, granulocyte colony‐stimulating factor, inositol‐phosphate metabolism, epidermal growth factor receptor, and somatostatin signaling. Literature reports confirm the relevance of these pathways in astrocytes, contribution to astrocytic functions, and potential to contribute to diseases of the CNS. This information is summarized in the form of short profiles in Figure S7, Supporting Information.

We also surveyed the literature for reports indicating the relevance of these preferentially knocked out pathways/processes for rAAV transduction. Indeed, we found that two of the preferentially knocked out astrocyte‐associated pathways (inositol metabolism and epithelial growth factor receptor (EGFR) pathway) have also been associated with various processes during transduction with rAAV vectors, in particular with intracellular trafficking and stability of rAAV capsids.^[^ ^58^, ^59^ ^]^ Taken together, these results provide evidence for the involvement of several astrocyte‐relevant signaling pathways in transduction with rAAV9P1.

## 3 Discussion

Astrocyte‐targeting gene transfer vectors are equally required for basic research on CNS functions and the development of gene therapies for the treatment of major neurological diseases. The manifold advantages of rAAV vectors make them potent candidates, but their success will ultimately depend on the availability of well‐characterized, highly cell‐selective rAAV vectors as well as a thorough understanding of the cellular molecules and pathways enabling vector targeting.

Capsid engineering by insertion of peptides is a widely used approach to modify organ and cell selectivity of rAAV vectors.^[^ ^15^, ^60^, ^61^ ^]^ rAAV9P1 is a derivative of rAAV9 that contains an artificial peptide sequence (P1 = **RGD**LGLS) at the variable region VIII of the capsid known to be favorable for engineering.^[^ ^62^, ^63^, ^64^, ^65^ ^]^ Capitalizing on our previous discovery of rAAV9P1 as an astrocyte‐targeting vector in experiments with human neural cell cultures,^[^ ^26^ ^]^ we provide evidence that rAAV9P1 is capable of targeting astrocytes in the brains of mice after i.v. injection and thus retains astrocyte‐targeting functions across species. In addition, we used rAAV9P1 to elucidate cellular factors that enable targeting of astrocytes by rAAV vectors.

A detailed understanding of the cellular interaction molecules of engineered rAAV vectors with a distinct cellular tropism is fundamental for the progressive development of novel and increasingly selective vectors. While there are manifold variants of engineered rAAV vectors published, only a few studies could successfully identify distinct target molecules for the investigated rAAV vectors. A prominent example is the discovery of LY6A as a key factor for the strong neuro‐tropism and BBB penetrance of the rAAV variant rAAVPHP.B.^[^ ^66^, ^67^, ^68^ ^]^ Knowledge from such studies can be utilized for the design of next‐generation vectors equipped with receptor‐specific linker molecules such as nanobodies or designed ankyrin repeat proteins (DARPins) which will increase the possibility for selective transduction.^[^ ^69^, ^70^ ^]^

### 3.1 rAAV9P1 Targets Murine Astrocytes In Vivo

While our current and previous experiments revealed that rAAV9P1 showed astrocyte targeting and selectivity for human astrocytes over neurons in cell culture experiments, we could now confirm that rAAV9P1 also transduces astrocytes in the mouse CNS after systemic injection. In intact tissues, this vector excelled by transducing ≥ 90% astrocytes in the analyzed cortical areas when combined with a ubiquitous CMV promoter. Furthermore, we demonstrated that rAAV9P1 can also transduce astrocytes in a murine stab wound model indicating that astrocyte targeting in mice is not limited to intact tissues. In agreement, publicly available expression data from several studies confirmed that reactive astrocytes from various brain injury models of mice retain expression of the receptors of rAAV9P1 identified in this study, i.e., the integrins *α*v*β*3, *α*v*β*5, and *α*v*β*8, as well as the AAVR receptor. Given that there are various reports in which the in vitro transduction patterns of rAAVs do not necessarily translate to animal models,^[^ ^37^ ^]^ this validation of rAAV9P1 in the mouse model is very important in underlining its usefulness and value as a gene transfer vector in vivo. It has to be mentioned that the overall number of transduced CNS cells, especially in intact tissues, was relatively low in our experiments. This might be partly attributed to a robust transduction of different musculature including skeletal muscle, heart, and diaphragm by rAAV9P1 following peripheral delivery, as described for several synthetic rAAV vectors containing the RGD motif, including rAAV9P1 (also known as AAVMYO).^[^ ^65^, ^71^ ^]^ Further optimization of the rAAV9P1 vector will thus be necessary to prevent its dilution through transduction and gene expression outside the CNS and testing of other ubiquitous promoters is needed to reveal how specific the targeting is. To further restrict expression to astrocytes, future studies may also explore the use of different and potentially astrocyte‐specific promoters. However, this approach will require detailed analysis of cell‐specificity of gene delivery and expression, which may be influenced by alterations in promoter‐capsid interactions of the vectors^[^ ^72^ ^]^ as well as the stringency of promoter activities in various astrocyte subtypes.

### 3.2 Targeting of Astrocytes by rAAV9P1 Vectors is Mediated by Multiple Receptor Molecules

Numerous viruses interact with RGD‐binding integrins, including Adenoviruses, which are co‐infectants and helper viruses of natural AAV serotypes, as well as members of the family of *Herpesviridae* (e.g., EBV, HHV‐6, and CMV), Foot‐and‐mouth disease virus (FMDV), Coxsackie Virus, and possibly also SARS‐CoV‐2.^[^ ^73^ ^]^ In the light of the integrin‐binding motif RGD in the peptide displayed on the rAAV9P1 capsid, we hypothesized that RGD‐binding integrins on human astrocytes are important astrocytic interactors of the rAAV9P1 capsid. Indeed, we were able to identify key RGD‐binding integrins used by rAAV9P1 for transduction of astrocytes by evaluating transduction efficiencies of rAAV9P1 in astrocytic cell lines, combined with knockouts of various integrin genes and competition of RGD‐binding integrins with specific ligands in WT cells during exposure to vector. Collectively, our results demonstrate that *α*v*β*8 is an indispensable interactor of rAAV9P1 capsids for targeting of astrocytes since astrocyte transduction was eliminated by knockout of *α*v (*ITGAV*) or *β*8 (*ITGB8)* genes and strongly impaired by treatment of cells with the *α*v*β*8‐ligand 2a. The P1 peptide in rAAV9P1 contains a Leucine (L) residue proximal to the RGD motif (**RGDL**GLS). This L residue is critical for the interaction of ligands with *α*v*β*8, as was shown for *α*v*β*8‐ligand 2a^[^ ^52^ ^]^ and the latent transforming growth factor *β*, ligand of *α*v*β*8. This suggests that the L residue following the RGD motif in the rAAV9P1 vector is important for its binding to *α*v*β*8.^[^ ^74^ ^]^ In support, we have previously found that an rAAV9 variant containing the sequence RGDA instead of RGDL (rAAV9P3) transduced human astrocytic cells much less efficiently than rAAV9P1.^[^ ^26^ ^]^

Our study also reveals that the *α*v*β*3 and *α*v*β*5 integrin heterodimers are important for rAAV9P1 transduction of astrocytes. Supporting evidence provided here is that transduction of human astrocytic cells with rAAV9P1 was inhibited by treatment with a peptide ligand (CGT) with high binding affinities for *α*v*β*3 and *α*v*β*5.^[^ ^48^ ^]^ In addition, double‐knockout of the subunits *β*3 and *β*5 strongly reduced transduction. In contrast to the *β*8 knockout, inhibition of transduction was not complete, indicating that *β*3 and *β*5 integrins are important for astrocyte transduction but are not absolutely irreplaceable. Interestingly, single knockouts of either *β*3 or *β*5 did not affect transduction efficiencies, suggesting that *β*3 and *β*5 subunits may be able to substitute for each other in mediating the transduction of astrocytes by rAAV9P1.

Other rAAV vectors, particularly rAAV2 and rAAV9, have also been reported to engage RGD‐binding integrins such as *α*v*β*5, *α*v*β*3, and *α*5*β*1 for cell surface attachment and virus uptake.^[^ ^75^, ^76^, ^77^, ^78^ ^]^ The interaction of these capsids with the RGD‐binding integrins was mapped to an NGRmotif, conserved in the capsid sequences of rAAV9P1, rAAV9, and rAAV2 (position 511–513 in the VP3 protein of AAV2). The observed failure of rAAV9 vectors to efficiently target astrocytes (Figure 1) indicates that the NGR motif in the AAV9 capsid is not sufficient to enable integrin interactions for efficient transduction of human astrocytes. Nevertheless, in combination with the dominant integrin‐binding motif RGD in the P1 peptide, the NGR motif could play a supporting role, possibly by increasing the overall interaction of the rAAV9P1 capsid with *α*v*β*3 integrins and/or promoting internalization of capsids, together with AAVR.^[^ ^25^ ^]^

Our results indicate that efficient transduction of astrocytes with rAAV9P1 requires multiple RGD‐binding integrin receptors, with *α*v*β*8 acting as major receptor and *α*v*β*3 and *α*v*β*5 as ancillary receptors. RGD‐binding integrins are involved in many functions in healthy brains^[^ ^79^, ^80^, ^81^, ^82^ ^]^ and may be expressed at higher levels in injured brains^[^ ^81^ ^]^ and astrocytic tumors.^[^ ^83^, ^84^, ^85^ ^]^ Our study further emphasizes the importance of RGD‐binding integrins in the CNS by demonstrating their relevance as receptors of astrocyte‐targeting rAAV vectors.

Publicly available data on protein and RNA expression in *The Human Protein Atlas* indicate that not only astrocytes express the RGD‐binding integrins identified as interaction partners of rAAV9P1 in this study.^[^ ^86^ ^]^ The expression of RGD‐binding integrins in various cell types and tissues throughout the whole organism is not surprising. Given their role as key players in cellular functions such as adhesion, communication, and migration, it can be hypothesized that these integrins, either individually or in combination, might also be involved in the transduction of other cell types, including various muscle cell types, by rAAV9P1. A detailed analysis of the interaction of rAAV9P1 with integrins on other cell types can be expected to further advance our understanding of the transduction mechanism of this vector, but is beyond the scope of this study.

While our results clearly show that integrin interactions mediated by the P1 peptide are essential for astrocyte‐targeting of rAAV9P1, we also demonstrate that astrocyte targeting requires the interaction of the rAAV9P1 capsid with additional known AAV receptors. These include both glycan and proteinaceous receptors. Concerning glycan receptors, we found that rAAV9P1 capsids, like the parental rAAV9, use N‐linked terminal galactose, presumably for attachment,^[^ ^54^, ^87^ ^]^ and are inhibited by the presence of sialic acids on cell surface glycans. Interestingly, transduction of astrocytes by rAAV9P1 was independent of HSPG, in contrast to rAAV2 capsids which could be completely prevented from transducing astrocytes by soluble heparin. Furthermore, rAAV9P1 lacks the arginine residues (R585 and R588) critical for HSPG interaction of AAV2.^[^ ^24^, ^45^, ^88^ ^]^ Since HSPGs are integral components of the surface of many different cell types,^[^ ^89^ ^]^ HSPG‐independent transduction of astrocytes by rAAV9P1 may be instrumental for higher selectivity of rAAV9P1 for astrocytes over other cell types. In support of this, elimination of HSPG binding activities of AAV2 has been reported to lead to rAAV2 variants with higher cell selectivity and increased tissue tropism.^[^ ^90^, ^91^, ^92^, ^93^, ^94^ ^]^

For proteinaceous AAV receptors, we show that transduction of astrocytes by rAAV9P1 and rAAV2 depends on AAVR, as expected.

**Figure 8:**
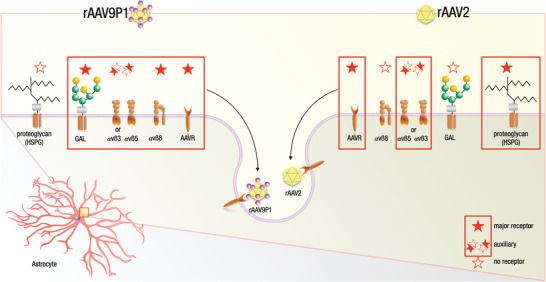
Overview of receptors used by rAAV9P1 and rAAV2 vectors for transduction of human astrocytes. The results of this study indicate that the receptor signature used by rAAV9P1 for transduction of human astrocytes consists of the major receptors integrin *α*v*β*8, AAVR, and N‐linked proteoglycans with terminal galactose, as well as the auxiliary receptors integrin *α*v*β*3 or *α*v*β*5. In contrast, major receptors for transduction of human astrocytes by rAAV2 vectors comprise AAVR and HSPG, with *α*v*β*3 or *α*v*β*5 as possible (weak) auxiliary receptors. Filled red stars indicate major receptors, half‐filled stars auxiliary receptors, and empty stars indicate no receptor function. The major and auxiliary receptors composing the receptor signature pattern of rAAV9P1 (left) and rAAV2 (right) are boxed in red.

Overall, our results show that rAAV9P1 uses multiple cell‐surface receptors for the transduction of human astrocytes. This is summarized in **Figure** **8**, which illustrates receptor usage of rAAV9P1 for transduction of human astrocytes, in comparison to rAAV2. In this model, rAAV9P1 capsids attach to cell‐surface proteoglycans with N‐linked terminal galactose and interact with a set of proteinaceous receptors comprising the RGD‐binding integrins *α*vß8 and *α*v*β*3 or *α*v*β*5, and AAVR. The interactions of rAAV9P1 with the RGD‐binding integrins and/or AAVR may facilitate uptake of the virus capsid since *α*v*β*5 and AAVR have both been reported to contribute to internalization of AAV2 capsids.^[^ ^25^, ^58^, ^76^ ^]^

To obtain an even more comprehensive picture of astrocytic genes contributing to successful transduction with rAAV9P1 vectors, we complemented our hypothesis‐guided analysis of rAAV9P1 receptors with an unbiased CRISPR/Cas9 genome‐wide knockout screening to evaluate cellular pathways and biological processes required for transduction of astrocytes by rAAV9P1. The resulting gene list consisted predominantly of genes encoding proteins with intracellular location and yielded nine preferentially knocked out pathways. A more detailed literature study revealed four pathways and one biological process highly relevant for astrocytes (see Figure S7, Supporting Information). These pathways or biological processes in astrocytes contribute to key functions in the CNS, including synaptic functioning, regulation of the BBB, development of brain cells, neuron‐astrocyte communication, and cognitive performance (Sonic hedgehog signaling,^[^ ^95^, ^96^ ^]^ G‐CSF‐induced signaling,^[^ ^97^, ^98^, ^99^, ^100^, ^101^, ^102^, ^103^ ^]^ inositol phosphate metabolism,^[^ ^104^, ^105^, ^106^ ^]^ EGFR,^[^ ^107^, ^108^ ^]^ somatostatin signaling^[^ ^109^, ^110^, ^111^ ^]^).

Furthermore, these pathways are involved in various diseases of the brain, including neuroinflammation, Alzheimer's disease, ischemic stroke, and tumors. Interestingly, two of these pathways are also associated with intracellular events important for AAV infection, further supporting the relevance of astrocyte‐related pathways for AAV transduction. Overall, our results raise the possibility that several pathways with strong astrocyte relevance might facilitate post‐entry processing of rAAV9P1 in astrocytes and thereby contribute to efficient transduction of astrocytes with rAAV9P1. These results form the basis for future hypothesis‐driven investigation of the contributions of individual pathways to efficiency and selectivity of rAAV9P1 astrocyte‐targeting and of their roles for targeting different astrocyte subtypes, especially in association with CNS diseases.

## 4 Outlook and Conclusion

To our knowledge, this is the first study addressing cellular components of human astrocytes involved in targeting by rAAV vectors. The present study was made possible by the prior identification and characterization of rAAV9P1 as an astrocyte‐targeting vector using cultured human neural cells.^[^ ^26^ ^]^ Demonstration of superior astrocyte‐targeting capabilities of rAAV9P1 compared to rAAV9 and rAAV2 vectors and proof‐of‐concept for targeting of astrocytes in vivo establish rAAV9P1 as a prototypical astrocyte‐targeting rAAV vector. This makes rAAV9P1 an exciting tool for research of many aspects of human astrocytes, including astrogenesis, drug susceptibilities, and contributions of astrocytes to major diseases of the CNS.

This initial study lays the groundwork for many further investigations. In particular, the multi‐receptor pattern delineated for rAAV9P1 will facilitate the development of advanced next‐generation astrocyte‐targeting vectors and accelerate the development of detargeting strategies. Concurrently, the biological pathways and processes identified by genome‐wide CRISPR/Cas9 screening in U251MG cells open multiple avenues for hypothesis‐driven investigation of cellular pathways contributing to the successful targeting of astrocytes with rAAV vectors.

We conclude that the analysis of the interactions of rAAV9P1 with cellular constituents of astrocytes reported in this study will support astrocyte research and, ultimately, the development of novel vector‐based strategies for therapy of key diseases of the brain involving astrocytes.

## 5 Experimental Section

### Cell Culture

All cell lines were cultured at 37 °C and 5% CO_2_. Human cell lines U251MG (astrocytic glioblastoma), Hek293T (human embryonal kidney), and HeLa (human epithelial) were cultured in Dulbecco's modified Eagle medium (DMEM) supplemented with 10% fetal calf serum (FCS) and 1% antibiotic‐antimycotic (all Gibco). The human fetal neural stem cell line HNSC.100^[^ ^112^ ^]^ is a neural progenitor cell line that gives rise to terminally differentiated astrocytes upon growth factor removal. Successful differentiation of HNSC.100 cells to astrocytes was addressed extensively and was demonstrated by differentiation‐associated upregulation of astrocyte markers including *gfap* and *aquaporine 4*, increase in astrocyte‐like morphology,^[^ ^113^, ^114^ ^]^ and discontinued proliferation.^[^ ^26^ ^]^ Details of the culture of proliferating HNSC.100 cells (HNSC.100 prol) and differentiation conditions were reported before.^[^ ^26^ ^]^

### rAAV Production

Production of recombinant scAAV (rAAV) vectors was performed as described earlier.^[^ ^26^, ^115^ ^]^ For production of rAAV2‐eYFP, rAAV9P1‐eYFP,^[^ ^26^ ^]^ rAAV9‐eYFP, or the rAAVP1‐eYFP variants used in cell culture experiments, AAV helper plasmids encoding the AAV2 *rep* gene and the *cap* gene of the respective serotype or variant were used. AAV capsid variants S1P1, S10P1, H15P1, and D20P1 were generated in two steps, by first selecting a shuffled library composed of AAV1, AAV6, AAV8, and AAV9 in the musculature of mice, and by then engrafting the P1 peptide onto selected variants with high muscle specificity. Details of the in vivo selection and helper plasmid cloning strategies are reported elsewhere (El Andari et al., manuscript in revision). The transfer plasmid pBSUF3‐YFP and pHelper^[^ ^29^ ^]^ were used for all vector productions. rAAV vectors were produced on medium scale by triple‐transfecting 10 × 15 cm dishes of HEK293T cells using polyethylenimine (PEI; Polyscience) and purified by iodixanol density gradient as reported previously.^[^ ^116^ ^]^ rAAV vectors were stored in aliquots of 50 µL in iodixanol (40% in PBS + 1 × 10^−3^ m MgCl_2_, 2.5 × 10^−3^ m KCl) at −80 °C.

For rAAV injections in vivo, vectors were produced on large scale by triple‐transfecting 60 × 15 cm dishes of HEK293T cells using PEI and purified using cesium chloride (CsCl) density gradient centrifugation. The detailed protocol was reported before.^[^ ^117^ ^]^

For rAAV vector titration by quantitative polymerase chain reaction (qPCR), vector capsids were lysed by mixing vector stocks (10 µL) with TE buffer (10 µL; Tris‐HCl (1 × 10^−3^ m; Sigma‐Aldrich), EDTA (0.01 × 10^−3^ m; Carl Roth, pH 8), and NaOH (20 × 10^−3^ m; Carl Roth)). Samples were incubated at 56 °C for 30 min and neutralized by addition of HCl (38 mL, 1 m; Carl Roth). Lysates from iodixanol purified vector preparations were diluted 1:1000 in MilliQ water (1:100 for CsCl‐purified vector preparation) and 2 µL was used for each reaction. Virus titers were determined by absolute quantification as vector genomes per milliliter (vg mL^−1^) by interpolation from a standard curve.

For transduction, rAAV vectors were thawed and directly added to the cell culture medium at an MOI of 10 000 if not indicated otherwise. All rAAV vectors contained either an *eyfp* or *gfp* transgene under the control of a cytomegalovirus (CMV) promoter. In all qPCRs, primers eYFP forward and eYFP reverse were used (Table S1, Supporting Information).

### Flow Cytometry

Cells were harvested, washed twice in PBS + 1% FCS, and analyzed using a FACSCanto II flow cytometer and the FACSDiva software (BD Bioscience). Analysis and evaluation of the raw data were performed using the FlowJo V10 software (FlowJo, LLC). In general, flow cytometry analysis was performed for three independent transduction experiments with two or three replicates per experiment. The live‐cell population was defined by cell size and granularity as determined by forward (FSC) and side scatter (SSC), gated for eYFP measurement in the FITC‐channel, and plotted against SSC. Background fluorescence was determined using untransduced samples and gates for eYFP‐positivity were set accordingly. Transduction rates were determined as percentage of eYFP‐positive cells out of at least 10 000 events in the live gate. For detection of surface staining (see the Supporting Information for details), mean fluorescence intensities of the live cell population were determined and depicted relative to unstained controls or controls stained with the secondary antibody only. Between cell lines, FSC, SSC, as well as the “live” gate were adjusted to the properties of the respective cell type.

### Conditions for Assaying Inhibition/Modulation of rAAV Vector Transduction in Wildtype Cells

Cells were grown overnight in 96‐well or 48‐well format and treated and/or transduced the next day under the following conditions.

For integrin inhibition, U251MG or HNSC.100 cells were co‐incubated with the indicated and nontoxic concentrations of CGT (Tocris Bioscience) or *α*v*β*8‐ligand 2a and rAAV9P1‐eYFP or rAAV2‐eYFP for 48 h at 37 °C. Alternatively, fully differentiated HNSC.100 cells in 48‐well plates were used in this experiment.

For heparin competition, U251MG cells were co‐incubated with soluble heparin (1, 2, or 4 mg mL^−1^; neoFroxx) and rAAV9P1‐eYFP or rAAV2‐eYFP for 4 h at 37 °C. Cells were washed with pre‐warmed PBS, supplied with fresh DMEM, and incubated for 48 h at 37 °C.

For modulation of the surface glycan landscape, U251MG cells were treated with neuraminidase (Neu; 50 mU) from *Vibrio cholerae* (Merck‐Millipore) for 2 h at 37 °C. Subsequently, cells were chilled on ice for 5 min, the supernatant was removed, and cells were co‐incubated with *Erythrina cristagalli* lectin (ECL)‐biotin (10 µg mL^−1^; Vector Laboratories) and rAAV9P1‐eYFP or rAAV2‐eYFP for 1.5 h on ice. Cells were washed thrice with ice‐cold PBS, supplied with fresh DMEM, and incubated at 37 °C for 48 h. Alternatively, cells and bound virus particles were directly lysed in 50 mL direct lysis buffer and used in an rAAV binding assay (rABA).

After the 48 h incubation period, the expression of the *eyfp* transgene was measured by flow cytometry. In all experiments, untreated and/or untransduced cells were served as controls.

### Animals and Tail Vein Injections

Animal handling and experimental procedures were performed in accordance with German and European Union animal welfare policies and were approved by the State of upper Bavaria (Identification number: ROB‐55.2‐2532.Vet_02‐17‐151). All efforts were made to minimize suffering and number of animals. Mice were maintained in specific pathogen‐free conditions housed in groups of two to three animals in filter top cages with 12 h:12 h light:dark cycles.  Mice had free access to water (acidified and desalinated) and standard rodent chow (Altromin, 1310M). Both adult male and female mice, between 2 and 4 months of age, were used for the experiments. Tail vein injections were performed as previously described.^[^ ^118^ ^]^ Briefly, rAAV9P1 vector preparations corresponding to 2–3 × 10^12^ viral genomes were resuspended in saline (150–200 µL) and injected in the tail vein. Three different preparation batches were used. Batch 1 and 3 were two different preparations of rAAV9P1‐CMV‐eYFP, while batch 2 was rAAV9P1‐CMV‐GFP. Two mice were injected with batch 1 and three mice were injected with batch 3. Three mice were injected with batch 2 and received a unilateral cortical stab wound 2 days before the tail vein injection as previously described.^[^ ^119^ ^]^ In these mice, the analysis was performed in the intact contralateral hemisphere. To study rAAV9P1 tropism in the injured brain, a cortical stab wound was performed in the motor cortex 3 days before transduction with viral vectors, as previously described.^[^ ^120^ ^]^ A report of the animal groups and the viral batches used is provided in Table S3 in the Supporting Information. Brain sections were prepared, stained, and analyzed as described in the Supporting Information.

### CRISPR/Cas9‐Mediated Knockout Generation in U251MG Cells

U251MG knockout cell lines were generated using a CRISPR/Cas9‐mediated gene targeting approach as described earlier.^[^ ^121^ ^]^ In detail, one sgRNA (20‐mer) targeting an early exon‐coding region of each respective gene was chosen (detailed sequences are listed in Table S1, Supporting Information)^[^ ^122^ ^]^ and cloned into the pMini‐U6‐sgRNA‐CMV‐Puro‐T2A‐Cas9 backbone using a ligase‐independent cloning technique.^[^ ^123^ ^]^ U251MG cells were transfected with pMini‐U6‐sgRNA‐CMV‐Puro‐T2A‐Cas9 (500 ng) using Lipofectamine 2000 stem cell transfection reagent (Invitrogen) according to the manufacturer's manual. Cells were selected in medium containing puromycin (0.8 µg mL^−1^) for 3 days and plated under limiting dilution conditions. Identified grown clones were replated and the presence of desired genomic knockouts was verified by deep sequencing (Illumina's MiSeq platform). Only clones carrying bi‐allelic frameshift mutations were picked and used for further experiments. U251MG knockouts with attachment deficits (U251MG *ITGAV* ^−/−^, U251MG *ITGB5* ^−/−^, U251MG *ITGB3/B5* ^−/−^) were grown on flasks and plates pre‐coated with 1% collagen.

### Genome‐Wide CRISPR/Cas9 Knockout Screen

U251MG‐Cas9 cells were generated by lentiviral transduction with lentiCas9‐blast^[^ ^56^ ^]^ and subsequently re‐seeded in medium containing blasticidin (20 mg mL^−1^; InvivoGen) for selection until untransduced control cells had died under selection pressure. To generate the CRISPR KO library, 7 × 10^7^ U251MG‐Cas9 cells were transduced with lentivirus of the human GeCKO v2 A sublibrary,^[^ ^56^ ^]^ 65383 sgRNA sequences targeting 19050 protein‐coding genes plus 1000 nontargeting control sgRNAs) at an MOI of 0.4 and subsequently re‐seeded in medium containing 0.8 µg mL^−1^ puromycin (Sigma‐Aldrich) for selection. A total of 1 × 10^7^ mutagenized cells were collected for genomic DNA extraction to assess the sgRNA representation of the starting populations. For the screen, 3 × 10^7^ U251MG‐Cas9_GeCKO cells were transduced with rAAV9P1‐eYFP at an MOI of 100 000 for 48 h. A second set of 3 × 10^7^ cells remained untransduced as control. Transduced cells were sorted for eYFP‐negativity on a BD FACSAria III flow cytometry cell sorter (BD Bioscience), using an eYFP‐negative gate that was determined beforehand using untransduced control cells. Genomic DNA was isolated from sorted eYFP‐negative cells and unsorted control cells by standard phenol/chloroform extraction.

The sgRNA expression cassettes were amplified from genomic DNA in a two‐step PCR using Phusion High‐Fidelity DNA polymerase (Thermo Scientific). For PCR1, 32 reactions (for control samples) and three to five reactions (for eYFP‐negative sorted samples) containing genomic DNA (5 µg) were set up and amplified for 18 cycles. Reactions were pooled, mixed, and cleaned up using NucleoSpin Gel and PCR cleanup kit (Machery and Nagel). For PCR2, seven reactions containing PCR1 (10 µL) products were amplified for 18 cycles using indexed primers. PCR products were purified using NucleoSpin Gel and PCR cleanup kit (Machery and Nagel) and sequenced at PrimBio. Primer sequences are listed in Table S1, Supporting Information.

The sgRNA sequences identified in the sequencing data from the different populations were matched to their respective protein‐coding genes. Downstream analyses were performed with the best‐performing sgRNA per gene that yielded the most reads. The enrichment of sgRNAs in the eYFP‐negative sorted population in comparison to the unsorted control population was calculated as a fold increase of sgRNA reads. This resulted in an initial set of 2715 individual genes (see Table S2a and Figure S6, Supporting Information). To increase the biological significance of the results, the minimum number of reads for an sgRNA to be considered in the comparison was set to 100 and the lower threshold of fold increase was set to twofold. This final set of 1326 genes (Table S2b, Supporting Information) was subjected to pathway, tissue, gene ontology (GO‐), and medical subject headings (MeSH‐) Term analysis using the GeneRanker tool (Genomatix AG). The results were ranked according to *p* values (0 < 0.99 e‐3) and shown at their rank position (Figure 7B).

### Statistical Analysis

All statistical tests were performed by using the two‐way ANOVA unless stated otherwise. In the case of the influence of two different factors, Sidak's correction for multiple comparisons was employed. If only one factor was influencing the result, a one‐way analysis of variance (ANOVA) test was applied. Data are represented as mean ± SEM of three independent transduction experiments (*n* = 3) with two technical replicates each. Independent transduction experiments were performed with cells of different passages and the same virus preparation, if possible. For the analysis of tail vein injection experiments in mice (*n* = 13), a one‐way ANOVA and Kruskal–Wallis test were employed as indicated in the figure legend. In all cases, significance was defined as *p* ≤ 0.05. GraphPad Prism 9.0.2 software (GraphPad Software Inc.) was used for all statistical analyses.

## Conflict of Interest

D.G. is a co‐founder of the company AaviGen GmbH. D.G. and J.E.A are inventors on a pending patent application (International application number: PCT/EP2019/060790; Publication number: WO/2019/207132) covering AAVMYO and P1 peptide.

## Author Contributions

A.B. designed and performed all experiments that involve rAAV vector production, transduction of cell lines, and particle modeling. M.P. performed in vivo injections and performed sectioning, staining, and fluorescence microscopy of brain samples. M.G. supervised in vivo experiments and interpreted data; D.N. and V.H. provided plasmids for knockout experiments and performed sequencing and genotyping of knockout cell lines; J.A.S. and T.W. analyzed sequencing data from the CRISPR/Cas9 knockout screen; A.K. took electron microscopy pictures; H.K. provided the *α*v*β*8‐ligand 2a; J.E.A. cloned and provided helper plasmids for production of the additional P1‐displaying capsids studied in this work and assisted in flow cytometry analysis of their transduction efficiencies; D.G. provided the plasmids for rAAV vector production, supervised vector production and made major contributions to the interpretation of the data and the manuscript design; R.B.‐W. (Principal investigator) conceived and designed experiments and was responsible for finalizing the manuscript. All authors contributed to the finalization of the manuscript.

## Supporting information

Supporting Information

Supplemental Table 1

Supplemental Table 2a

Supplemental Table 2b

Supplemental Table 3

## Data Availability

The data that support the findings of this study are available from the corresponding author upon reasonable request.
