# Supplementary material for: Molecular Signature of Astrocytes for Gene Delivery by the Synthetic Adeno‐Associated Viral Vector rAAV9P1
Source: Adv Sci (Weinh). 2022 Apr 10;9(16):2104979. doi: 10.1002/advs.202104979 (PMC9165502; doi:10.1002/advs.202104979)
Supplement: Supplementary file 1 — Supporting Information [file ADVS-9-2104979-s003.pdf]

ADVANCED SCIENCE
Open Access

Supporting Information

for *Adv. Sci.*, DOI 10.1002/advs.202104979

Molecular Signature of Astrocytes for Gene Delivery by the Synthetic Adeno-Associated Viral Vector rAAV9P1

*Amelie Bauer, Matteo Puglisi, Dennis Nagl, Joel A Schick, Thomas Werner, Andreas Klingl, Jihad El Andari, Veit Hornung, Horst Kessler, Magdalena Götz, Dirk Grimm and Ruth Brack-Werner**

Supporting Information

Molecular signature of astrocytes for gene delivery by the synthetic adeno-associated viral vector AAV9P1

*Amelie Bauer, Matteo Puglisi, Dennis Nagl, Joel Anton Schick, Thomas Werner, Andreas Klingl, Jihad El Andari, Veit Hornung, Horst Kessler, Magdalena Götz, Dirk Grimm, Ruth Brack-Werner**

Quantitative real-time PCR:

Quantitative real-time PCR (qRT-PCR) was performed for absolute quantification of rAAV titers or analysis of bound virus particles after rABA. In both cases, a serial dilution of the AAV vector plasmid served as an external standard for absolute quantification. qPCR was conducted using the LightCycler® FastStart DNA Master SYBR Green I-Kit (Roche Diagnostics) according to the manufacturer's protocol on a Roche LightCylcer® 480 II system with standard LightCycler software (Roche Diagnostics). Primer sequences are listed in Table S1.

Cell titer blue (CTB) viability assay:

U251MG or HNSC.100 were grown overnight in black 96-well plates with clear bottoms and treated with the respective inhibitor for 48 h. CTB reagent (Promega) was thawed and mixed 1:5 with the respective culture medium. Culture supernatant was discarded from the plates and diluted CTB reagent (65 µl) was added to each well. Six wells were left without medium and served as "dead cell" controls. Cells were incubated with CTB reagent at 37 °C for one to three hours depending on the cell type, or until the supernatant in untreated control cells turned from blue to violet. The "dead cell" control wells were supplemented with 65 µl CTB reagent and the CTB assay was analyzed in an Infinite M200 microplate reader (Tecan, Excitation wavelength 550 nm, Emission wavelength 600 nm). Viability was determined relative to untreated controls, with untreated controls being considered 100 %.

Cell surface staining:

U251MG WT or integrin subunit knockout cells were grown in 96-well plates until full confluency. Cells were detached from the plate using EDTA (2 mM; Roth) in PBS and

transferred to a 96-well V-bottom plate. Cells were washed once with FACS buffer (PBS + 1 % FCS (Gibco)) and incubated in primary antibody diluted in FACS buffer (50 µl) for 30 min on ice in the dark. The following primary antibodies were used: mouse anti-human αvβ3 (5 µg/10^6 cells), anti-human αvβ5 (2.5 µg/10^6 cells), anti-human integrin β1 (0.25 µg/10^6 cells), anti-human integrin β8 (1 µg/10^6 cells), or anti-human αv AlexaFluor 647 (1 µg/10^6 cells, all from R&D systems). Cells were washed twice with FACS buffer and, if required for labelling, incubated with secondary antibodies (50 µl goat anti-mouse AlexaFluor 488 (Invitrogen, 1:200)), for 30 min on ice in the dark. Two final washing steps were conducted and cells were resuspended in FACS buffer (200 µl) and analyzed by flow cytometry analysis.

Electron microscopy:

For the analysis via transmission electron microscopy (TEM), samples were applied to carbon-coated copper grids and negatively stained with 2 % uranyl acetate as described previously.[1] After air drying, the samples were investigated at 80 kV with a Zeiss EM 912, equipped with an integrated OMEGA filter in the zero-loss mode. Images were acquired using a 2k x 2k CCD camera (TRS Tröndle Restlichtverstärkersysteme) with the respective software package.

Brain sectioning and immunostaining:

Mice were anesthetized and trans-cardially perfused first with cold phosphate buffer saline (PBS) and then 4% paraformaldehyde (PFA) in PBS. Brains were post-fixed for 24 h in 4% PFA at 4 °C and then cut using a vibratome (50 µm thick sections). Brain slices were incubated in blocking solution containing 3% bovine serum albumin and 0.5 % Triton X-100 for 30 min minimum and then with primary antibodies diluted in blocking solution over night at 4 °C, in agitation. The following primary antibodies were used: chicken anti-GFP (dilution 1:1000, Aves Labs), mouse anti-NeuN (dilution 1:250, Merck Millipore), rabbit anti-SOX9 (dilution 1:1500, Merck Millipore). The next day, sections were washed three times in PBS for 10 min and then incubated with the appropriate secondary antibodies diluted in blocking solution for two hours at room temperature and in agitation. The following secondary antibodies were used: donkey anti-chicken AlexaFluor 488 (dilution 1:1000, Dianova), goat anti-mouse IgG1 AlexaFluor 647 (dilution 1:1000, ThermoFisher), donkey anti-rabbit Cy3 (dilution 1:1000, Jackson ImmunoResearch). Finally, sections were washed three times with

PBS, stained with 4’, 6-diamidino-2-phenylindole (DAPI) for 10 min, and finally washed again three times in PBS before mounting.

Quantification and statistical analysis:

After immunostaining, confocal images were used to count and quantify the transduced cells. Micrographs (6-15 μm thick z stacks) of the mouse cerebral cortex spanning from the white matter to the pial surface were acquired with a confocal laser scanning (Zeiss LSM710) microscope. Three micrographs of the motor and barrel cortex per mouse were acquired and analyzed using the ImageJ software (NIH). The presented results represent an average of the results from six sections from different areas analyzed per mouse. Cellular tropism was determined by counting the percentage of GFP^+ or YFP^+ cells expressing Sox9 or NeuN. Cellular morphology was also considered to distinguish between astrocytes and neurons. Absolute numbers of neurons and astrocytes were calculated by counting the numbers of reporter positive cells per mm^3. The normality of the distribution of data points was verified using Shapiro-Wilk test. For data sets having normal distribution, statistical significance was calculated using one-way ANOVA followed by Bonferroni post-test. When normality tests failed to show normal distribution, non-parametric Kruskal-Wallis and Dunn’s multiple comparison tests or Mann-Whitney U test were used as indicated in the figure legends. ns $p>0.05$, * $p<0.05$, ** $p<0.01$, *** $p<0.001$.

Analysis of the expression of rAAV9P1 receptors in human brain cells, murine brain cells, and different cell lines in publicly available datasets:

Murine brain cells: gene expression data of rAAV9P1 receptors in intact and injured murine brain tissue was extracted from the two publicly available Affymetrix Mouse 430 2.0 Array datasets GSE66370 [2] and GSE35338.[3] Raw data was downloaded, reanalyzed to calculate fold changes, and plotted using R version 4.1.1 and RStudio version 1.4.1717.

Human brain cells: gene expression data of rAAV9P1 receptors in sorted human brain cells was downloaded from a publicly available dataset.[4] Raw data was filtered, reanalyzed to calculate fold changes, and plotted using R version 4.1.1 and RStudio version 1.4.1717.

Human cell lines: protein expression data of rAAV9P1 receptors in different human cell lines was downloaded from the Expression atlas.[5] Absolute expression of receptors (in arbitrary expression units, AEU) were extracted for the two distinct cell lines U251MG and HeLa, as well as for all available cell lines with astrocytic or neuronal origin. As representatives, the astrocytic and neuronal cell lines showing the highest receptor expression among all analyzed

cell lines were chosen. Extracted expression values were plotted with the GraphPad Prism 9.0.2 software.

In vitro recombinant AAV-binding assay (rABA):

The rABA was conducted as described earlier with slight modifications.[6] Cells in one well per plate were harvested and counted to determine cell numbers. Cells in 48-well plates were pre-chilled on ice for 5 min and incubated with rAAV vectors for 30 min on ice. After incubation, cells were washed carefully with ice-cold PBS without detaching any adherent cells. After removal of PBS, cells were lysed in direct lysis buffer (50 µl; Tris (10 mM, Carl Roth), $MgCl_2$ (3 mM, Carl Roth), EDTA (1 mM, Carl Roth), $CaCl_2$ (1 mM, Carl Roth), 1 % Triton-X100 (Carl Roth), 20 mg/ml Proteinase K (VWR)) directly in the well and transferred to a 0.2 ml PCR tube. Lysis was conducted in a thermo-cycler for 1 h at 56 °C and Proteinase K was inactivated for 10 min at 95 °C. Lysates were stored at -20 °C until analysis by qPCR. For analysis by qPCR, 2 µl lysate were used for each reaction. Absolute number of vg (vector genomes) per cell were determined by interpolation from the standard curve and normalization to absolute cell number determined before cell harvest. In all qPCRs, the primers eYFP forward and eYFP reverse were used (Table S1).

Structural modeling and sequence comparison:

Alignment of the $VP3_{AAV9}$ and $VP3_{AAV9P1}$ amino acid sequences was performed with the T-coffee online tool (http://tcoffee.crg.cat/).[7] The coordinates of the $VP3_{AAV9P1}$ model were generated using wildtype AAV9 PDB coordinates (RCSB PDB [https://www.rcsb.org/], PDB model ID: 3UX1) as template in the Phyre2 online tool (http://www.sbg.bio.ic.ac.uk/~phyre2/html/page.cgi?id=index)[8]. Multimer assembly of AAV9P1 was generated using the SWISS-MODEL online tool (https://swissmodel.expasy.org/).[9,10] Cartoon representation of the VP3-monomers and 60-mer, structural alignments, and depth-cue surface representations were generated using the UCSF-ChimeraX software.[11,12]

SDS-PAGE and immunoblot analysis

Whole cell lysates were generated using lysis buffer (100 mM Tris-HCl pH 8.3, 150 mM NaCl, 1 % Glycerol, 1 % NP40, 0.5 % Deoxycholate, 0.1 % SDS, 0.1 % Triton X-100) supplemented with cOmplete™ protease inhibitors (Roche). The concentration of the isolated

proteins was determined using BCA Protein Assay Reagent (Pierce). Ten µg of total protein were mixed with 4x NuPAGE™ sample buffer (Invitrogen) and dH_2O to a total volume of 18 µl and heated to 95 °C for 10 min. Samples were separated on a 12 % polyacrylamide gel in TGS buffer (25 mM Tris, 200 mM glycine, 0.1 % SDS) and proteins were blotted onto a 0.45 µm nitrocellulose membrane. After protein transfer, membranes were blocked in 5 % milk in PBS-T (0.1% Tween 20) for 1 h at RT and incubated in mouse anti-KIAA0319L (Abcam, 1:500) diluted in 5 % milk in PBS-T overnight at 4 °C. The next day, the membranes were washed 3x 10 min in PBS-T and incubated with anti-mouse peroxidase (Jackson ImmunoResearch Laboratories Inc., 1:10,000) diluted in 5 % milk in PBS-T for 1.5 h at RT. The membrane was rewashed 3x 10 min in PBS-T. Before image acquisition, the membrane was overlaid with SuperSignal™ West Femto Substrate Kit (Invitrogen), incubated for 1 min in the dark, and the signal was acquired using a Fusion FX chemiluminescence detector.

Supporting Figures

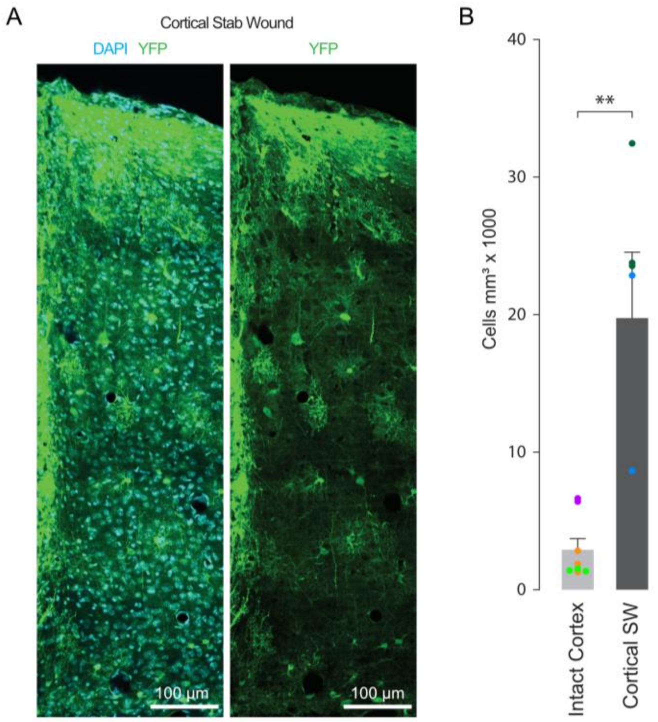

Figure S1. Transduction of stab wound lesion in mouse by rAAV9P1.

(A) Confocal micrographs showing an overview of the injured mouse motor cortex one month after the systemic injection of rAAV9P1. Stab wound injury was performed three days before the systemic injection. eYFP expression was detected using an anti-GFP antibody that cross-reacts with eYFP and images show YFP alone (right) or merged with a nuclear staining (DAPI, left). Scale bars represent 100 µm. (B) Histograms showing the number of eYFP-positive cells per area in the intact (left) or injured (right) mouse motor cortex after systemic injection of rAAV9P1. Circles represent individual animals and are color-coded according to separate experiments and virus batches as specified in Supporting Table S3. Data is presented as mean ± SEM (Intact cortex: n=8, cortical SW: n=5). p-values are calculated using Mann-Whitney U test. ns $p > 0.05$, ** $p \leq 0.01$.

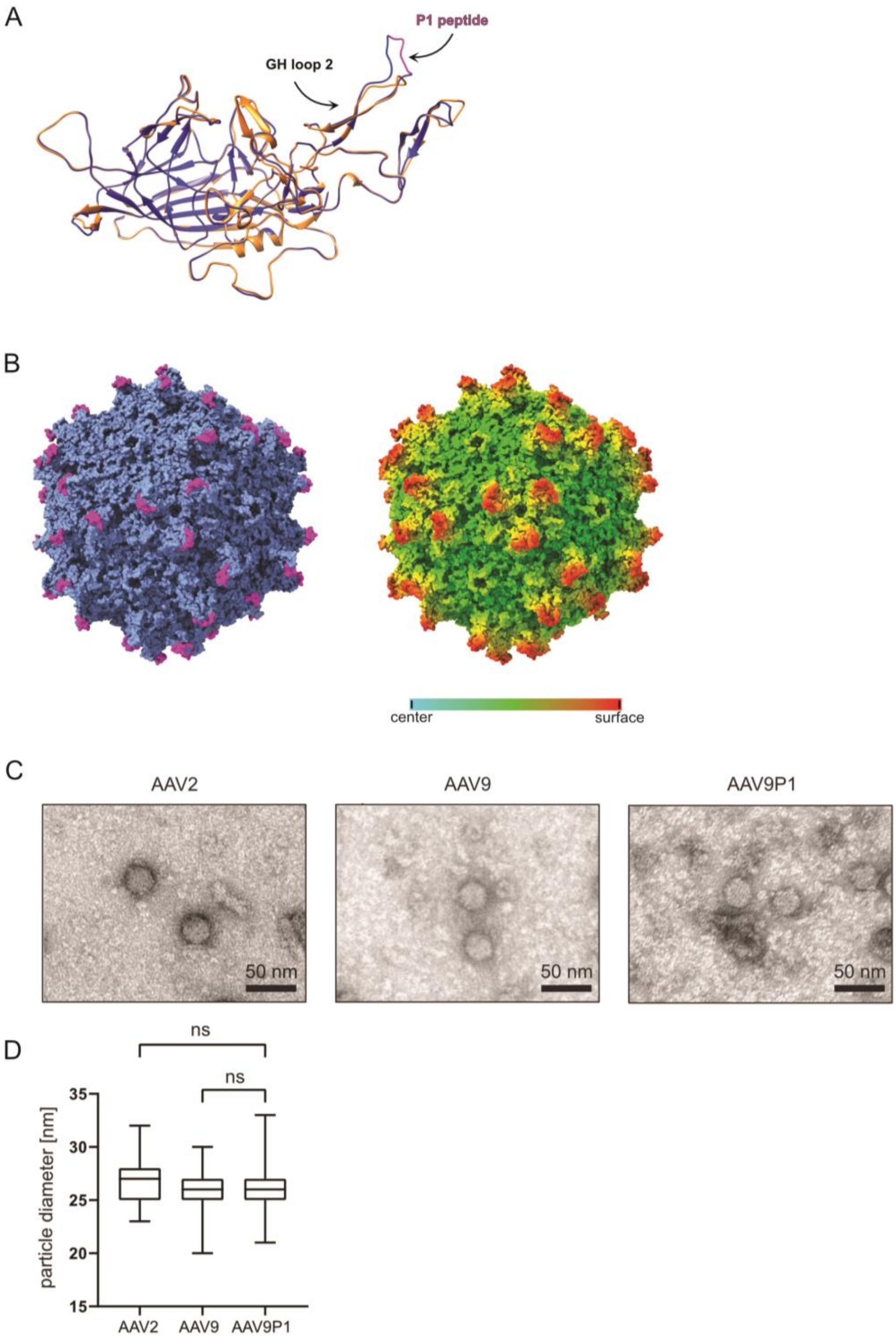

Figure S2. Localization of the P1 peptide within the VP3 monomer and assembled capsid and comparison of gross particle morphologies of rAAV2, rAAV9P1, and rAAV9 vectors.

(A) Structural alignment of the $VP3_{AAV9P1}$ (blue) and the $VP3_{AAV9}$ proteins (orange). Black arrows indicate localization of the P1 peptide (magenta, $VP1_{AAV9P1}$ aa 591-597) and of the VR-III ($VP1_{AAV9}$ aa 581-593). (B) *In silico* modelling of a fully assembled rAAV9P1 particle. Image shows surface localization of the P1 peptide in magenta (left) and coloring

according to surface depth (right). (C) Representative images of rAAV2, rAAV9, and rAAV9P1 particles in rAAV stocks obtained by transmission electron microscopy analysis. (D) Mean particle diameter of rAAV vector particles. Data depicts the mean diameters of 100 particles measured for each rAAV vector stock ± SEM. Two outliers were identified and removed from the dataset prior to statistical analysis by ROUT. p-values are calculated using one-way ANOVA with Šidák correction, ns $p > 0.05$.

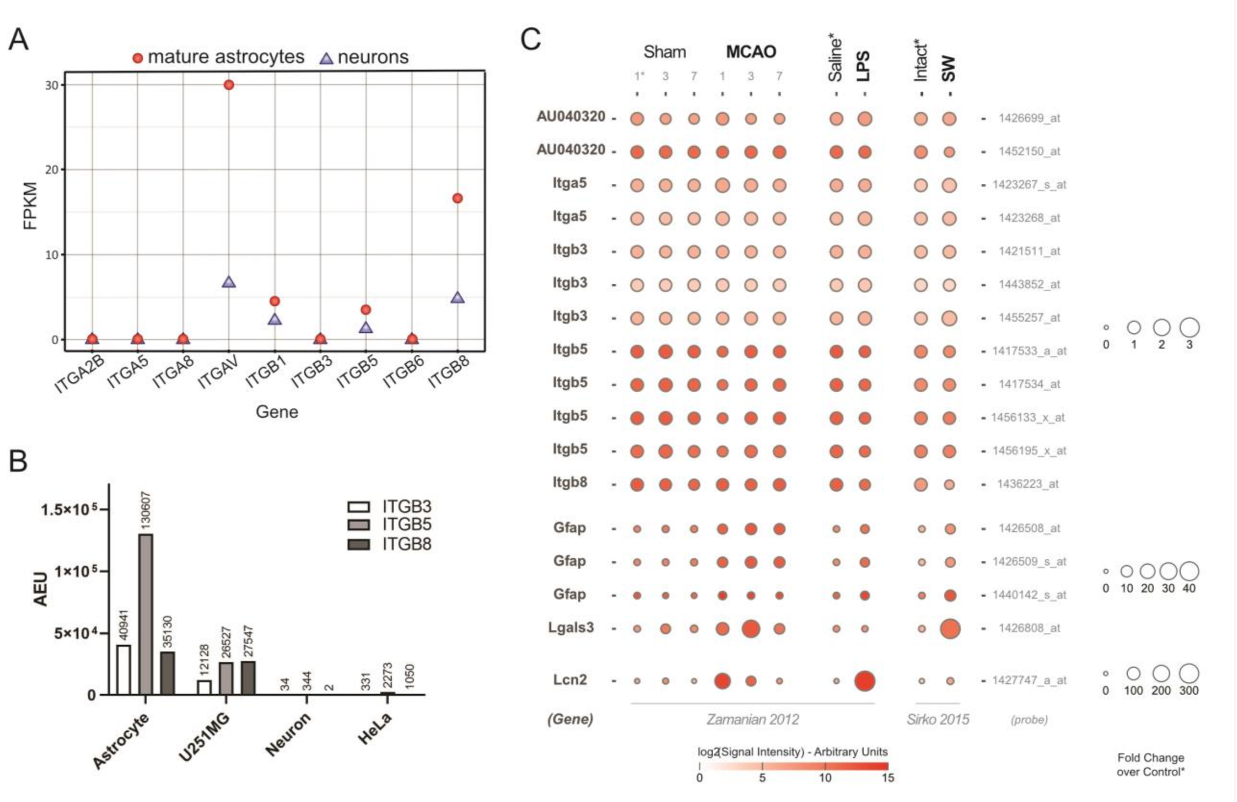

Figure S3. Analysis of publicly available data for expression of selected RGD-binding integrins by astrocytes. (A) Analysis of the differential RNA expression levels of selected integrin subunits that are known to form RGD-binding heterodimers in mature human astrocytes and neurons. This graph was generated by re-analysis of an online available RNA sequencing dataset from antibody-assisted cell sorting of human CNS cells published by Zhang et al.,[4] using anti-HepaCAM and anti-Thy1 antibodies, respectively, for separation of mature astrocytes and neurons. FPKM (fragments per kilobase per million mapped reads) depicts the relation between detected reads that are mapped to the respective gene and the total number of mapped reads in the library while also considering the gene length for normalization. (B) Protein expression of selected integrins by different human astrocytic and neuronal cell lines, and the HeLa cell line. The graph shows protein baseline expressions of the three integrins subunits ß3, ß5, and ß8 in arbitrary expression units (AEU) as taken from

the Expression atlas (https://www.ebi.ac.uk/gxa/home) for two multiple-cell-line examples (astrocyte and neuron) and two specific cell lines used in this study (U251 MG and HeLa). The values shown for the multiple-cell-line examples reflect the maximum reported expression values. The absolute expression value for each integrin subunit is indicated above the respective bar. (C) RNA expression levels of selected integrins in murine astrocytes populations isolated from healthy brain tissue and from various murine brain injury models. This graph was generated by re-analysis of two publicly available RNA sequencing datasets published by Zamanian et al.[3] and Sirko et al.[2] Expression level is defined by signal intensity (color coding) and fold-change over control (circle size). MCAO: ischemic induced by **m**iddle **c**erebral **a**rtery **o**cclusion; LPS: neuroinflammation resulting from systemic injection of lipopolysaccharide; SW: Stab wound. Uninjured brain tissues: Sham, Saline, Intact; Gfap, Lgals3, Lcn2: controls for astrocyte reactivity in injured condition; AU040320: murine homolog of KIAA0319L (AAVR).

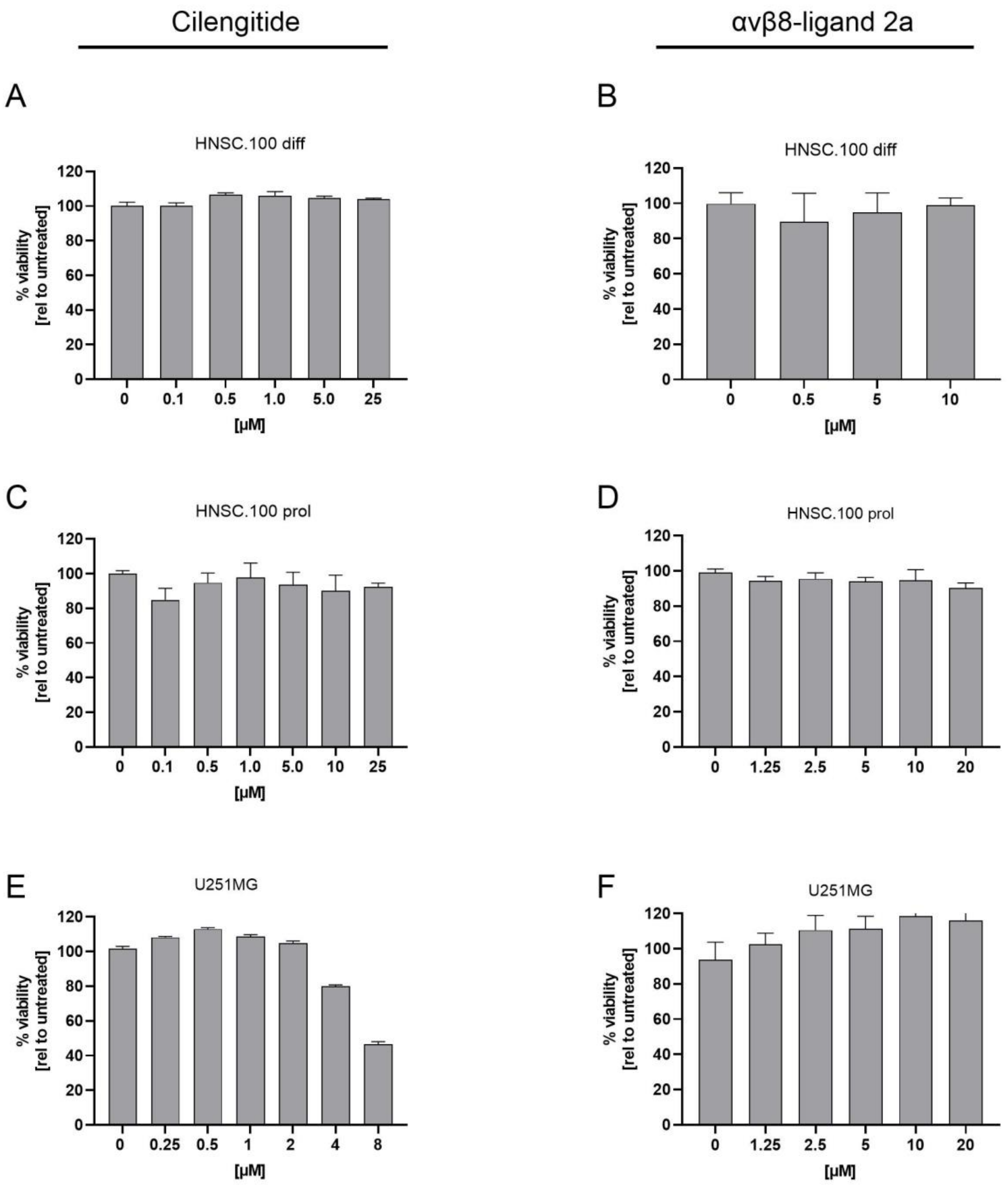

Figure S4. Assessment of cytotoxic effects of Cilengitide and αvβ8-ligand 2a treatment on different cell lines by Cell Titer Blue (CTB) viability test.

(A+C+E) Treatment of cells with Cilengitide (CGT). (B+D+F) Treatment of cells with αvβ8-ligand 2a. Cultures of differentiated HNSC.100 astrocytes (HNSC diff, A+B), proliferating HNSC.100 astrocyte progenitors (HNSC.100 prol, C+D) and U251MG astrocytic glioblastoma cells (E+F) were exposed to different concentrations of CGT (cRGDfNMeV) or αvβ8-ligand 2a (c(GLRGDLp(NMe)K(Ac)) and cell viability was determined by CTB cell viability assay 48 h after treatment. Data is depicted as mean ± SEM of two independent experiments (n=2) and is presented relative to vector-exposed, untreated control cells.

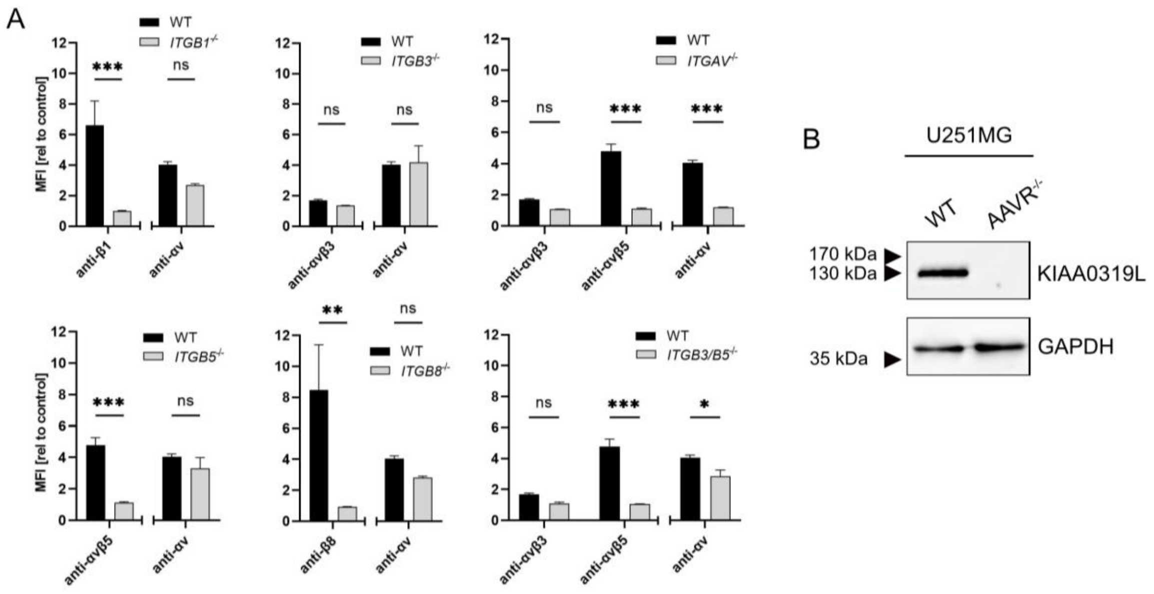

Figure S5. Analysis of U251MG knockout cell lines for expression of rAAV9P1 receptors by cell surface staining or immunoblot. All clones carry out-of-frame mutations of both alleles as identified by Illumina MiSeq sequencing. (A) Surface staining of knockout cell lines was performed with antibodies against assembled αvβ3 or αvβ5 heterodimers, or the subunits β1, β8, or αv. Samples stained with anti-αvβ3, anti-αvβ5, anti-β1, and anti-β8 antibodies were subsequently stained with a secondary anti-mouse Alexa Fluor® 488 antibody. Stainings with anti-αv Alexa Fluor® 647 did not require secondary staining. anti-αvβ3, anti-αvβ5, anti-β1, and anti-β8: data depicts median fluorescence intensity (MFI) of the FITC-channel relative to samples stained with anti-mouse Alexa Fluor® 488 only. anti-αv: data depicts MFI of the APC-channel relative to unstained samples. Data is presented as mean ± SEM of two independent stainings (n=2). p-values are calculated using two-way ANOVA with Šidák correction. ns $p > 0.05$, * $p \leq 0.05$, ** $p \leq 0.01$, *** $p \leq 0.001$. (B) Lysates of U251MG wildtype (WT) and $AAVR^{-/-}$ cell lines were run on 12 % polyacrylamide gels under denaturing conditions. After transfer to a 0.45 µm nitrocellulose membrane, the blotted bands were immunodetected with a specific anti-KIAA0319L or anti-GAPDH antibody and subsequently visualized with peroxidase labelled goat anti-mouse antibody.

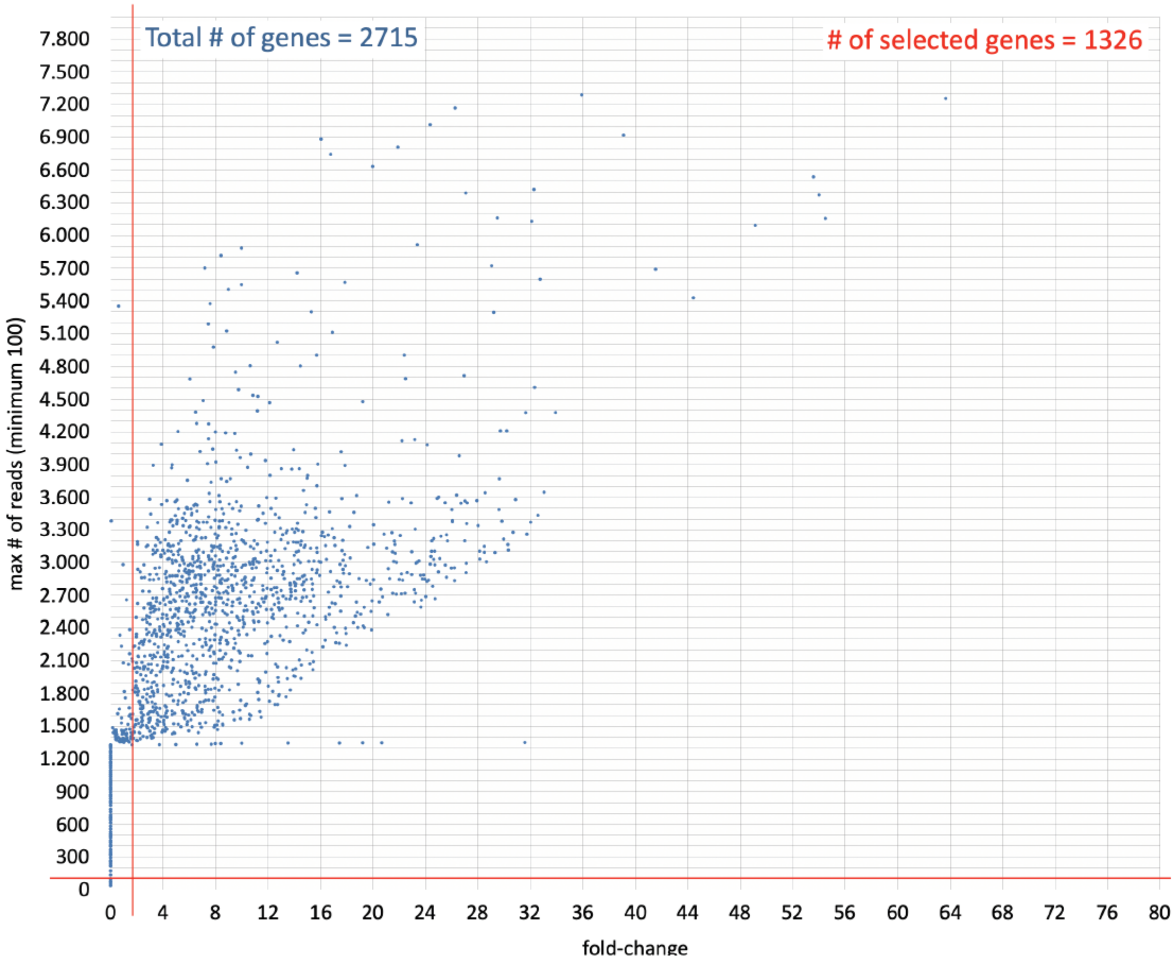

Figure S6. Scatterplot of genes detected in the CRISPR/Cas9 knockout screening. The graph depicts the number of sgRNA reads detected for the respective gene in the eYFP-negative sorted population (y-axis) and their expression fold-change relative to the unsorted control population (x-axis). The red lines indicate the cut-off parameters chosen for biological relevance (≥ 2.0 fold-change of sgRNAs, ≥ 100 reads), yielding a total of 1,326 genes for further analysis.

Sonic hedgehog (SHH) signaling

Sonic hedgehog is generated by neurons and is a well-known regulator of neurodevelopment. Multiple roles for SHH signaling in astrocytes have also been identified in the mature brain. Thus SSH-signaling in astrocytes is important for astrocyte-dependent modulation of synaptic functions and regulation of neuronal activity, limitation of astrocyte mediating inflammation and for promoting BBB integrity.
(References: [13,14])

G-CSF-induced signaling:

The cytokine G-CSF (granulocyte colony-stimulating factor) exerts neuroprotective as well as neuroinflammatory effects and is an important determinant of brain health. Evidence for G-CSF-induced signaling in astrocytes has been reported in the context of many diseases of the CSF. Thus, expression of G-CSF receptors by astrocytes was shown in ischaemic stroke, Alzheimer's disease, and multiple other CNS diseases. G-CSF was also reported to be a survival growth factor for glioblastomas and can inhibit glial scar formation by astrocytes.
(References: [15–21])

Inositol phosphate metabolism:

Inositol 1,4,5-triphosphate (IP3) is a second messenger that triggers Ca^{2+} release from the ER. IP3-Ca^{2+} signaling in astrocytes is important for numerous astrocytic functions, including extracellular ion (K+) homeostasis, synaptic glutamate clearance, and neurotransmitter release by astrocytes. IP3-Ca^{2+} signaling in astrocytes is important for cognitive performance and maintenance of mental health and contributes to neuronal plasticity, learning, and memory. On the other hand, IP3-Ca^{2+} signaling in astrocytes has also been implicated in various disease conditions, including Alzheimers' disease, epilepsy, depression, ischemic stroke, and Alexander disease.
(References: [22–24])

EGFR (epidermal growth factor receptor):

EGFR regulates the development of astrocytes from neural progenitor cells and the maintenance of neural stem cell pools. Furthermore, it is important for the formation of astrocyte processes, which are essential for neuronal support.

Concerning the injured brain, EGFR is involved in astrocyte reactivation and astrogliosis and influences glial scar formation in injured brains.
Overexpression of EGFR in astrocytes is observed in Alzheimer's disease and EGFR amplification is a diagnostic criterium for certain types of aggressive diffuse astrocytic glioblastomas.
(References: [25,26])

Somatostatin signaling:
Somatostatin is released by neurons. Somatostatin-driven astrocyte signaling enhances Ca2+ signaling in astrocytes. It is involved in the communication of GABA-ergic interneurons with astrocytes and contributes to the balancing of excitatory and inhibitory synaptic functioning. Somatostatin receptor expression on astrocytes is increased in Alzheimers brains.
(References: [27–29])

Figure S7. Short profiles of astrocyte-relevance of preferentially knocked out pathways. This figure contains summaries of literature information demonstrating astrocyte association of preferentially knocked out pathways/biological processes identified by the genome-wide CRISPR/Cas9 screening.

Supporting Tables legends

Table S1. Oligonucleotides. This table contains all primer and oligonucleotide sequences used in this publication. All oligonucleotides were purchased from Metabion. Columns indicate name, sequence, and usage of the respective oligonucleotides. Oligonucleotides used for LIC assembly of vectors for CRISPR/Cas9 mediated knock-out generation include sgRNA sequences for the respective gene.

Table S2. CRISPR/Cas9 knock-out screen gene list. (A) List of all 2,715 preferentially knocked out genes detected in the eYFP-negative sorted population (Gene name, Gene ID), the read frequency of the four sgRNAs used per gene (#reads), the read frequencies of the respective sgRNAs in the unsorted control population, and fold-changes of the most efficient

sgRNA per gene in eYFP-negative sorted relative to unsorted control. (B) Filtered set of 1,326 genes (minimum number of reads set to 100 and the lower threshold of 2-fold increase of sgRNA reads).

Table S3. Additional information on vector injections in mice. This table contains information about the groups of animals used for individual experiments, including number of animals per group, color coding for Figure 2 and Figure S1, presence and type of injury, analyzed cortex area, and virus batch used.

References

[1] J. Kaletta, C. Pickl, C. Griebler, A. Klingl, R. Kurmayer, L. Deng, *Sci. Rep.* **2020**, *10*, 18625.

[2] S. Sirko, M. Irmler, S. Gascón, S. Bek, S. Schneider, L. Dimou, J. Obermann, D. De Souza Paiva, F. Poirier, J. Beckers, S.M. Hauck, Y.A. Barde, M. Götz, *Glia* **2015**, *63*, 2340.

[3] J.L. Zamanian, L. Xu, L.C. Foo, N. Nouri, L. Zhou, R.G. Giffard, B.A. Barres, *J. Neurosci.* **2012**, *32*, 6391.

[4] Y. Zhang, S.A. Sloan, L.E. Clarke, C. Caneda, C.A. Plaza, P.D. Blumenthal, H. Vogel, G.K. Steinberg, M.S.B. Edwards, G. Li, J.A. Duncan, S.H. Cheshier, L.M. Shuer, E.F. Chang, G.A. Grant, M.G.H. Gephart, B.A. Barres, *Neuron* **2016**, *89*, 37.

[5] Https://www.ebi.ac.uk/gxa/home, [accessed 17/02/2022]

[6] P. Guo, J. Zhang, M. Chrzanowski, J. Huang, H. Chew, J.A. Firrman, N. Sang, Y. Diao, W. Xiao, *Mol. Ther. - Methods Clin. Dev.* **2019**, *13*, 40.

[7] C. Notredame, D.G. Higgins, J. Heringa, *J. Mol. Biol.* **2000**, *302*, 205.

[8] L.A. Kelley, S. Mezulis, C.M. Yates, M.N. Wass, M.J.E. Sternberg, *Nat. Protoc.* **2015**, *10*, 845.

[9] A. Waterhouse, M. Bertoni, S. Bienert, G. Studer, G. Tauriello, R. Gumienny, F.T. Heer, T.A.P. De Beer, C. Rempfer, L. Bordoli, R. Lepore, T. Schwede, *Nucleic Acids Res.* **2018**, *46*, W296.

[10] M. Bertoni, F. Kiefer, M. Biasini, L. Bordoli, T. Schwede, *Sci. Rep.* **2017**, *7*, 1.

[11] T.D. Goddard, C.C. Huang, E.C. Meng, E.F. Pettersen, G.S. Couch, J.H. Morris, T.E. Ferrin, *Protein Sci.* **2018**, *27*, 14.

[12] E.F. Pettersen, T.D. Goddard, C.C. Huang, E.C. Meng, G.S. Couch, T.I. Croll, J.H. Morris, T.E. Ferrin, *Protein Sci.* **2021**, *30*, 70.

[13] S.A. Hill, M. Fu, A.D.R. Garcia, *Cell. Mol. Life Sci.* **2021**, *78*, 1393.

[14] A.D.R. Garcia, *Cells* **2021**, *10*, 1353.

[15] M. Hasselblatt, A. Jeibmann, B. Riesmeier, D. Maintz, W.R. Schäbitz, *Acta Neuropathol.* **2007**, *113*, 45.

[16] B.G. Xiao, C.Z. Lu, H. Link, *J. Cell. Mol. Med.* **2007**, *11*, 1272.

[17] M.A. Wheeler, I.C. Clark, E.C. Tjon, Z. Li, S.E.J. Zandee, C.P. Couturier, B.R. Watson, G. Scalisi, S. Alkwai, V. Rothhammer, A. Rotem, J.A. Heyman, S. Thaploo, L.M. Sanmarco, J. Ragoussis, D.A. Weitz, K. Petrecca, J.R. Moffitt, B. Becher, J.P.

Antel, A. Prat, F.J. Quintana, *Nature* **2020**, *578*, 593.

[18] R.P. Revoltella, M. Menicagli, D. Campani, *Cytokine* **2012**, *57*, 347.

[19] S. Ridwan, H. Bauer, K. Frauenknecht, K. Hefti, H. Von Pein, C.J. Sommer, *J. Anat.* **2014**, *224*, 377.

[20] H. Chu, Y. Tang, Q. Dong, *Neuroscience* **2014**, *260*, 59.

[21] J.K. Choi, S.Y. Park, K.H. Kim, S.R. Park, S.G. Lee, B.H. Choi, *BMB Rep.* **2014**.

[22] Y. Okubo, *J. Pharmacol. Sci.* **2020,** *144*, 83.

[23] A. Pinto-Duarte, A.J. Roberts, K. Ouyang, T.J. Sejnowski, *Glia* **2019**.

[24] N.A. Smith, L.K. Bekar, M. Nedergaard, *Neurochem. Res.* **2020**, *45*, 100.

[25] R. Romano, C. Bucci, *Cells* **2020**, *9*, 1887.

[26] D.J. Brat, K. Aldape, H. Colman, E.C. Holland, D.N. Louis, R.B. Jenkins, B.K. Kleinschmidt-DeMasters, A. Perry, G. Reifenberger, R. Stupp, A. von Deimling, M. Weller, *Acta Neuropathol.* **2018**, *136*, 805.

[27] U. Kumar, *Neuroscience* **2005**, *134*, 525.

[28] L. Mariotti, G. Losi, A. Lia, M. Melone, A. Chiavegato, M. Gómez-Gonzalo, M. Sessolo, S. Bovetti, A. Forli, M. Zonta, L.M. Requie, I. Marcon, A. Pugliese, C. Viollet, B. Bettler, T. Fellin, F. Conti, G. Carmignoto, *Nat. Commun.* **2018**, *9*, 82.

[29] S. Mederos, G. Perea, *Glia* **2019**, *67*, 1842.
